# Reversal of Synapse Degeneration by Restoring Wnt Signaling in the Adult Hippocampus

**DOI:** 10.1016/j.cub.2016.07.024

**Published:** 2016-10-10

**Authors:** Aude Marzo, Soledad Galli, Douglas Lopes, Faye McLeod, Marina Podpolny, Margarita Segovia-Roldan, Lorenza Ciani, Silvia Purro, Francesca Cacucci, Alasdair Gibb, Patricia C. Salinas

**Affiliations:** 1 Department of Cell and Developmental Biology, University College London, London WC1E 6BT, UK; 2 Department of Neuroscience, Physiology, and Pharmacology, University College London, London WC1E 6BT, UK

## Abstract

Synapse degeneration occurs early in neurodegenerative diseases and correlates strongly with cognitive decline in Alzheimer’s disease (AD). The molecular mechanisms that trigger synapse vulnerability and those that promote synapse regeneration after substantial synaptic failure remain poorly understood. Increasing evidence suggests a link between a deficiency in Wnt signaling and AD. The secreted Wnt antagonist Dickkopf-1 (Dkk1), which is elevated in AD, contributes to amyloid-β-mediated synaptic failure. However, the impact of Dkk1 at the circuit level and the mechanism by which synapses disassemble have not yet been explored. Using a transgenic mouse model that inducibly expresses Dkk1 in the hippocampus, we demonstrate that Dkk1 triggers synapse loss, impairs long-term potentiation, enhances long-term depression, and induces learning and memory deficits. We decipher the mechanism involved in synapse loss induced by Dkk1 as it can be prevented by combined inhibition of the Gsk3 and RhoA-Rock pathways. Notably, after loss of synaptic connectivity, reactivation of the Wnt pathway by cessation of Dkk1 expression completely restores synapse number, synaptic plasticity, and long-term memory. These findings demonstrate the remarkable capacity of adult neurons to regenerate functional circuits and highlight Wnt signaling as a targetable pathway for neuronal circuit recovery after synapse degeneration.

## Highlights

• Wnt signaling is required for synapse integrity in the adult hippocampus
• Dkk1 induces synapse loss and deficits in synaptic plasticity and long-term memory
• Dkk1 disassembles synapses by activating the Gsk3 and Rock pathways
• Synapse loss and memory defects are reversible by reactivation of the Wnt pathway

## Graphical Abstract

**Figure:**
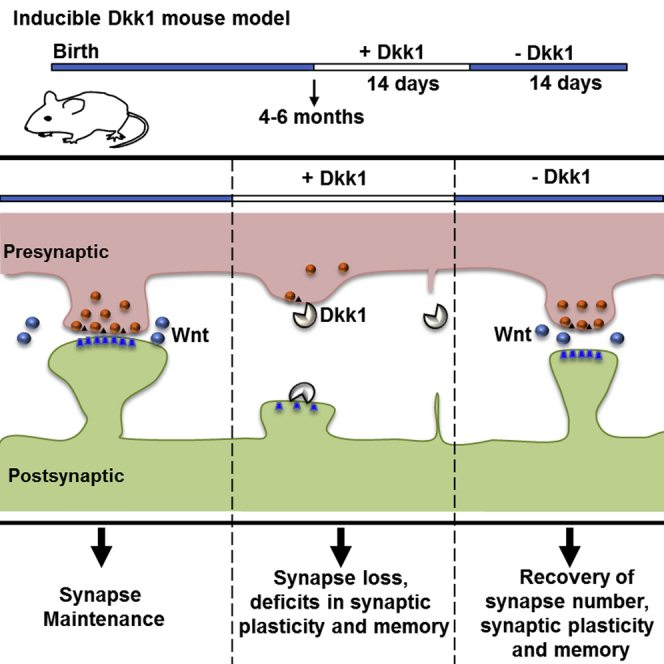

## Teaser

Deficiency in Wnt signaling has been implicated in Alzheimer’s disease. Marzo et al. elucidate the impact of the Wnt antagonist Dkk1 in the adult hippocampus, showing synapse loss and defects in synaptic plasticity and long-term memory. They also reveal that cessation of Dkk1 expression induces synapse regeneration and recovery of long-term memory.

## Introduction

Synapse loss and dysfunction are an early occurrence in several neurodegenerative conditions, including Alzheimer’s disease (AD). Synapse vulnerability strongly correlates with cognitive decline before detectable neuronal death [1, 2] and might contribute to the subsequent neuronal degeneration. Surprisingly, little is known about the molecular mechanisms that trigger synapse vulnerability in neurodegenerative diseases and even less about how this process can be prevented or reversed.

Increasing evidence suggests that deficient canonical Wnt signaling contributes to AD pathogenesis. Wnts are secreted proteins that modulate several aspects of brain development and function, including synapse formation, synaptic transmission, experience-mediated synaptic remodeling, and adult neurogenesis [3, 4, 5, 6, 7]. Genome-wide association studies (GWASs) have revealed a link between genetic variants of the Wnt co-receptor LRP6, which are associated with decreased canonical Wnt signaling activity, and late onset AD [8, 9]. Loss of function of LRP6 in hippocampal neurons results in synaptic defects, cell death, and exacerbation of amyloid deposition in a mouse model of AD [10]. Importantly, the secreted protein Dickkopf-1 (Dkk1), which blocks canonical Wnt-Gsk3 signaling by sequestering the LRP6 receptor [11, 12], is elevated in post-mortem brains from AD patients and in AD animal models [13, 14, 15]. In addition, oligomers of amyloid-β (Aβ), the main component of amyloid plaques in AD, induce Dkk1 expression in cultured neurons and in brain slices [13, 16, 17]. Dkk1 disassembles excitatory synapses in a similar manner to Aβ in cultured hippocampal neurons [17]. Importantly, blockade of Dkk1 with neutralizing antibodies protects synapses from Aβ-mediated disassembly [17]. Collectively, these results suggest that Dkk1-mediated deficiency of Wnt signaling could contribute to synapse vulnerability. However, the impact of Dkk1 on hippocampal circuits, which are severely affected in AD, and its mechanism of action have not been explored.

Restoration of synaptic function after substantial synapse loss is crucial for the treatment of neurodegenerative diseases, as diagnosis is often obtained after significant damage has occurred. Although some downstream targets of Aβ have been identified [18, 19, 20, 21], only a limited number of studies has shown the ability of these molecules to fully restore function after significant synapse degeneration [18, 20]. Thus, the identity of the signaling pathways that could restore synapse function remains poorly understood.

Here, we demonstrate a critical role for Wnt signaling in synapse stability and synaptic plasticity in the adult hippocampus. Using a transgenic mouse model that allows inducible expression of Dkk1, we investigated the contribution of deficient Wnt signaling to synapse function in the adult hippocampus without compromising embryonic and postnatal development. Inducible Dkk1 expression triggers disassembly of excitatory synapses, defects in long-term potentiation (LTP), and facilitation of long-term depression (LTD). Consistent with these synaptic plasticity changes, hippocampal-mediated long-term memory is impaired. These synaptic deficits occur in the absence of cell death or changes in the stem cell niche. Thus, the Dkk1 inducible (iDkk1) mouse is a good model system to study synapse degeneration in the absence of cell loss. Our studies reveal that Dkk1 induces synapse degeneration through the combined activation of Gsk3 and a novel target of Dkk1, the RhoA-Rock pathway. Notably, we found that reactivation of Wnt signaling, by cessation of Dkk1 expression, results in full recovery of synapse structure, synaptic plasticity, and long-term memory. In summary, our studies demonstrate that deficient Wnt signaling leads to synapse loss in vivo as observed at early stages of Aβ-mediated pathogenesis and reveal the remarkable regenerative capacity of neurons in the adult hippocampus to assemble synapses within functional circuits. Our work highlights the importance of Wnt signaling in this process and identifies new targetable molecules for protecting synapses from degeneration.

## Results

### Inducible Dkk1-Expressing Mice as a Model for Wnt Deficiency in the Adult Brain

**Figure 1:**
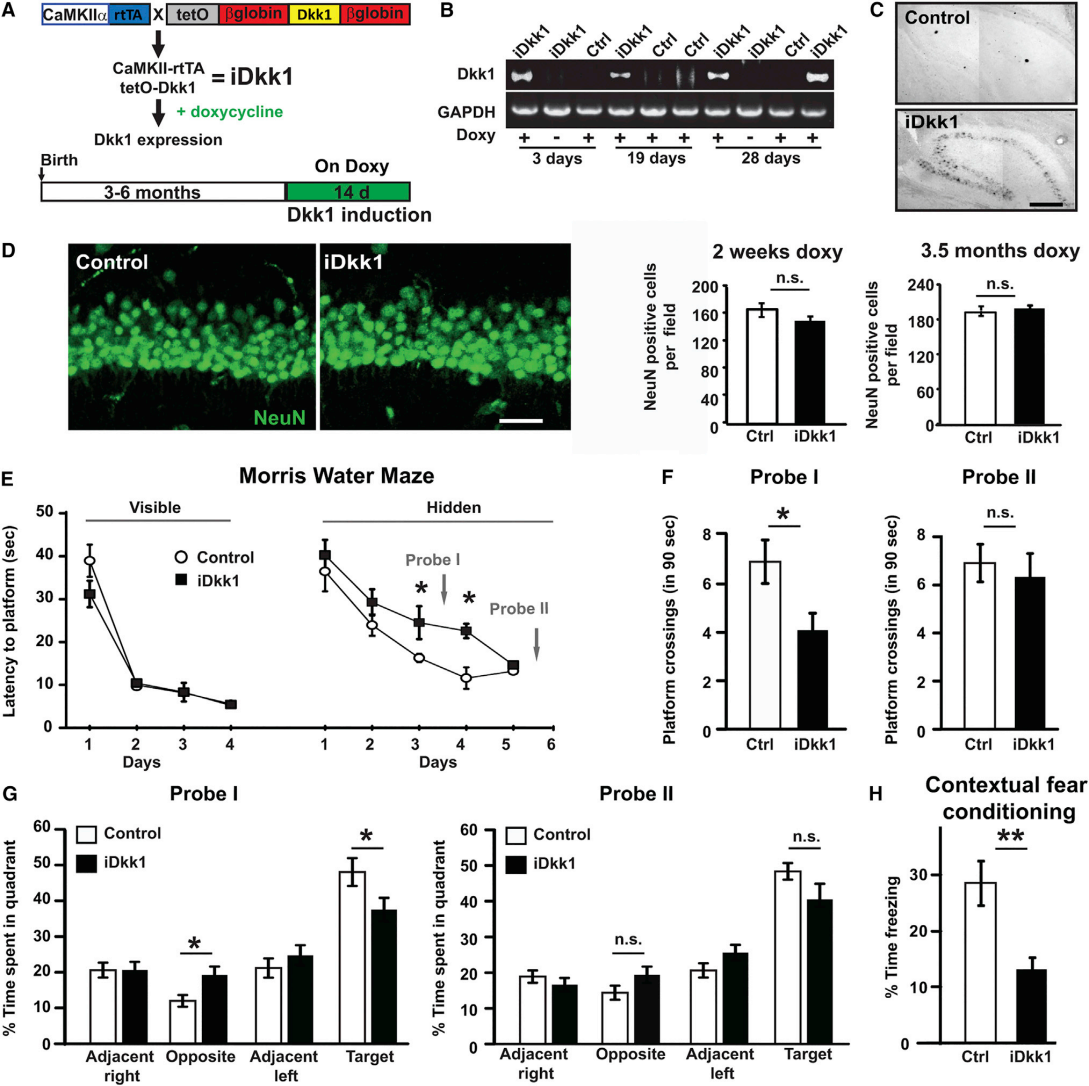
Expression of Dkk1 in Adult Hippocampus Induces Defects in Long-Term Memory (A) Top: schematic of doxycycline-induced Dkk1 expression. Mice carrying the rtTA gene under the CaMKIIα promoter are crossed with tetO-Dkk1 mice to generate double transgenic animals (iDkk1). Bottom: schematic representation of doxycycline feeding schedule in adult mice (see Figure S1). (B) RT-PCR for Dkk1 in hippocampus of adult iDkk1 and control mice with or without doxycycline administration (Doxy). (C) In situ hybridization for Dkk1 mRNAs in adult hippocampus of iDkk1 and control mice. The scale bar represents 250 μm. (D) Images and quantification of NeuN-positive CA1 neurons in control and iDkk1 mice fed with doxycycline for 14 days. Quantification of NeuN-positive CA1 neurons is also shown after 3.5 months of diet containing doxycycline (ANOVA; four mice per genotype per condition). The scale bar represents 50 μm (see also Figure S2). (E) Escape latency in the Morris water maze (MWM) (^∗^p < 0.05; repeated-measures ANOVA; 11 control and 12 iDkk1 mice; see also Figure S3). (F) Number of platform crossings in the MWM during probe I (left) or during probe II (right; ^∗^p < 0.05; Student’s t test). (G) Time spent in each quadrant during probe I (left) and during probe II (right; ^∗^p < 0.05; Student’s t test). (H) Percentage of freezing time evaluated 24 hr after the foot shock (^∗∗^p ≤ 0.01; ANOVA; eight control and seven iDkk1 mice). Data are represented as mean ± SEM.

To investigate the contribution of Wnt signaling to synapse maintenance in the adult hippocampus, we took advantage of a transgenic mouse model where expression of a potent and specific secreted Wnt antagonist, Dkk1, is controlled under the tetracycline-inducible system and CaMKII promoter [22]. Expression of Dkk1 is induced in adult mice by administration of doxycycline, bypassing any potential deleterious effects of deficient Wnt signaling during embryogenesis and postnatal development, stages when Wnt signaling plays a critical role [12, 23, 24, 25]. Mice carrying the Dkk1 coding region under the control of the doxycycline responsive element (tetO) [26] were crossed to mice carrying the tetracycline-controlled transactivator (rtTA2S; rtTA hereafter) downstream of the CaMKIIα promoter (CaMKII hereafter) [27]. Dkk1 expression was induced in adult (3–6 months of age) double transgenic mice (iDkk1) by administration of doxycycline into their diet for 2 weeks for full induction of the CaMKII-rtTA/tetO system [28] (Figure 1A).

Dkk1 expression was detected by RT-PCR in the hippocampus of adult iDkk1 mice fed with doxycycline, but not in control littermates fed with doxycycline or in iDkk1 mice not fed with doxycycline (Figure 1B). Dkk1 mRNA expression could be detected after 3 days of induction and sustained for the duration of doxycycline administration (Figure 1B). Thus, expression of Dkk1 is tightly regulated by doxycycline in iDkk1 mice. Most of our studies were performed after 2 weeks induction (unless otherwise indicated) when expression of Dkk1 was clearly observed by in situ hybridization (Figure 1C) in a large number of principal hippocampal neurons in the CA1, CA3, and dentate gyrus (DG). These mice developed normally and had similar weight to control mice (Figure S1).

### Dkk1 Does Not Affect Cell Death or the Stem Cell Niche in the Adult Hippocampus

Deficiency in Wnt signaling has been implicated in regulating cell viability and the stem cell niche in the adult hippocampus [13, 29, 30]. We therefore examined these two aspects in the hippocampus of adult iDkk1 mice expressing Dkk1 for 2 weeks. TUNEL assays and the levels of cleaved caspase 3 revealed no changes in cell death (Figures S2A–S2C). The number of NeuN-positive neurons was not altered (Figure 1D) after 14 days or after 3.5 months of Dkk1 induction. These findings demonstrate that induced Dkk1 expression in the adult hippocampus does not affect cell viability.

Next, we examined possible changes in the stem cell niche in the adult DG, the main source of neuronal stem cells in the hippocampus. The number of newly born neurons, labeled by the specific marker doublecortin (Dcx) [31], did not change upon Dkk1 induction (Figure S2D). Consistent with no changes in cell number, the overall morphology of the brain and the architecture of the hippocampus were normal (Figures S2A and S2B). Thus, induced expression of Dkk1 in the adult hippocampus does not affect cell viability or the stem cell niche.

### Wnt Signaling Blockade in the Adult Hippocampus Results in Long-Term Memory Deficits

The hippocampus plays a role in emotional and cognitive functions, such as anxiety, learning, and memory [32, 33]. We investigated the impact of Dkk1 expression in these processes. The exploratory activity and anxiety level, evaluated through an open-field and elevated plus maze, were identical in iDkk1 mice and controls (Figures S3A and S3B). In addition, no defects were observed in the swimming speed and traveled distance in a Morris water maze (MWM) (Figure S3C), demonstrating that Dkk1 expression does not affect motor function and hippocampal-dependent emotional behaviors.

Next, we investigated short-term memory using the discrete trial version of the spontaneous alternation T-maze test (30-s delay) [34]. This task depends on the animals’ natural tendency to alternate and enter the previously unvisited arm. Both control and iDkk1 mice alternated between the two arms above chance (Figure S3D), indicating that short-term memory is unaffected in iDkk1 mice. We then evaluated hippocampus-dependent spatial reference learning and long-term memory using the MWM test [35, 36, 37]. Mice were first trained on the cued version of the task (platform marked by a visible flag). No difference in the time required to reach the visible platform was observed between control and iDkk1 mice (Figure 1E), demonstrating that iDkk1 mice have no visual and procedural skills defects. Subsequently, mice were trained over 5 days to locate an invisible platform. The platform was removed during two probe tests (before the 4^th^ day and 24 hr after the 5^th^ day of training). iDkk1 mice took twice as long as controls to find the hidden platform on the 3^rd^ and 4^th^ days of training (Figure 1E), demonstrating an inability to remember the location of the platform. Similarly, during the first probe test (probe I), iDkk1 mice spent less time in the target quadrant and crossed the virtual platform location significantly fewer times than controls (Figures 1F and 1G), demonstrating impaired reference memory acquisition. Interestingly, after two further training days, iDkk1 mice reached the same performance level as control mice (probe II; Figures 1F and 1G), suggesting that additional training can overcome this memory deficit, as shown in some AD mouse models [38, 39, 40]. Thus, deficient Wnt signaling in the adult hippocampus leads to deficits in spatial memory acquisition.

To extend our study of memory-related hippocampal function, we used a single-trial contextual fear-conditioning paradigm [41, 42]. We compared the percentage of freezing time displayed by mice when re-introduced into the conditioning chamber after having associated the context to a foot shock. iDkk1 mice showed a considerably reduced freezing time compared to controls upon reintroduction to the conditioning chamber 24 hr after the context/shock single pairing (Figure 1H). This result indicates that iDkk1 mice were unable to form a strong association between the contextual cues and the foot shock. Together, our behavioral studies show that iDkk1 mice exhibit deficits in hippocampal-dependent long-term memory.

### Deficient Wnt Signaling Impairs Basal Synaptic Transmission and Synaptic Plasticity

**Figure 2:**
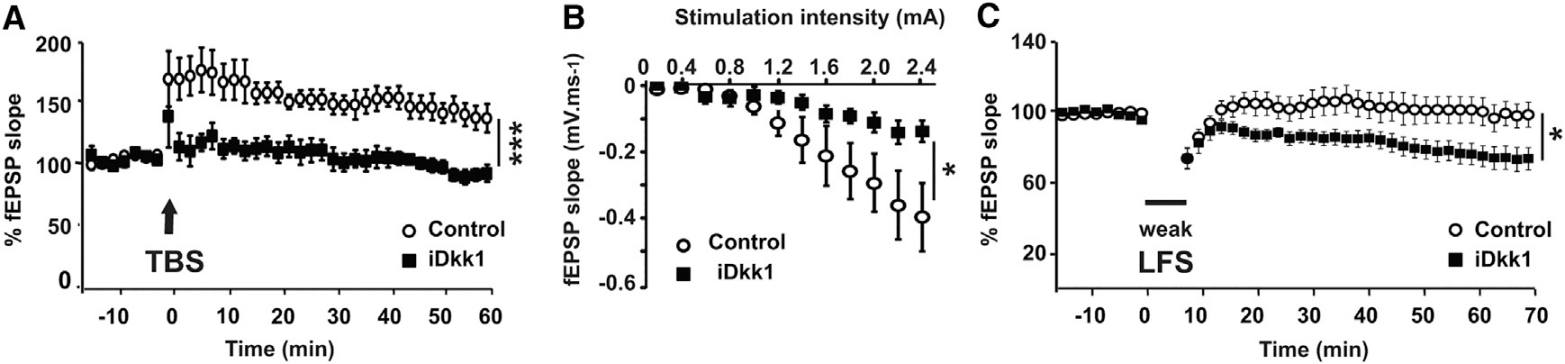
Dkk1 Impairs LTP and Basal Synaptic Transmission and Enhances LTD in the Adult Hippocampus (A) LTP was induced by theta-burst protocol (TBS) on Schaffer collateral axons (ten slices from six controls and eight slices from seven iDkk1 mice; ^∗∗∗^p ≤ 0.001; repeated-measures ANOVA). (B) Input-output curve shows fEPSP slope in CA1 in response to different stimulus intensity of Schaffer collateral axons (12 slices from eight control and 11 slices from seven iDkk1 mice; ^∗^p < 0.05; repeated-measures ANOVA). (C) A weak low-frequency stimulation (weak LFS) induces short-term depression in control slices and LTD in iDkk1 slices (11 slices from six controls and nine slices from five iDkk1 mice; ^∗^p < 0.05; repeated-measures ANOVA; see also Figure S4). Data are represented as mean ± SEM.

Changes in long-term memory have been correlated with changes in long-term synaptic plasticity (i.e., LTP and LTD) [43, 44, 45]. We therefore investigated the ability of iDkk1 mice to express LTP at Schaffer collateral (SC)-CA1 synapses. A theta-burst stimulation (TBS) protocol was chosen as it mimics hippocampal activity during spatial learning [46]. TBS induced a 40% potentiation in control mice, whereas in iDkk1 mice it failed to potentiate these synapses (Figure 2A), demonstrating that Wnt blockade in the adult hippocampus results in the absence of TBS-induced LTP.

This defect could be due to a decreased connectivity, as a minimal number of synapses is required to promote LTP induction as defined as cooperativity [47]. Analyses of input-output curves at the SC-CA1 synapses revealed a defect at the strongest intensities of stimulation in iDkk1 mice, as the field excitatory postsynaptic potential (fEPSP) slope reached only half the magnitude of control animals (Figure 2B). Thus, CA1 synaptic connectivity is affected by Dkk1 expression.

LTD is crucial to synaptic function, and its modulation by Wnt signaling remains unknown. To examine the impact of Dkk1 on LTD, we used a protocol that effectively induces LTD in adult mice with a strong low-frequency stimulation (LFS) consisting of two trains of 900 pulses at 2 Hz. With this protocol, we observed a 20%–30% depression at the SC-CA1 synapses in both control and iDkk1 animals (Figure S4). We therefore decided to use a sub-threshold LFS (weak LFS) protocol, which has been shown to unmask enhanced LTD after exposure to Aβ [48, 49]. We used a weak LFS protocol, consisting of a single train of 900 pulses at 2 Hz, which induced a short-term but no long-term depression in control animals (Figure 2C) [50]. In contrast to control animals, this weak LFS induced LTD in iDkk1 mice (Figure 2C). This is the first demonstration that Wnt signaling contributes to LTD expression. Thus, Wnt deficiency induced by Dkk1 expression facilitates LTD and blocks LTP at SC-CA1 synapses in the adult hippocampus.

### Dkk1 Triggers Degeneration of Excitatory Synapses in the Adult Hippocampus

**Figure 3:**
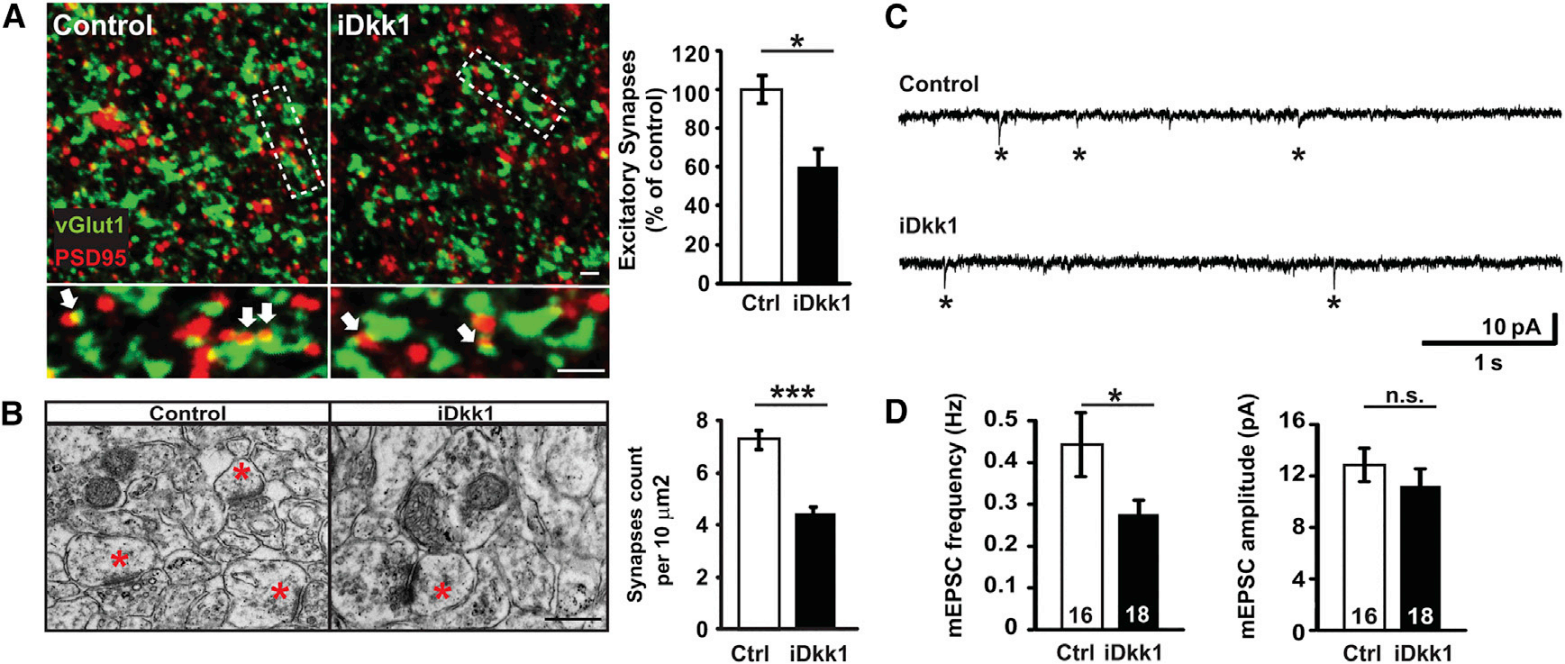
Blockade of Wnt Signaling Triggers Excitatory Synapse Loss and Dysfunction in the Adult Hippocampus in iDkk1 Mice (A) Confocal images from hippocampal CA1 show excitatory synapses (co-localized pre- and postsynaptic markers; vGlut1 and PSD95 puncta, respectively; white arrows). The scale bars represent 2 μm. Quantification is shown on the right-hand side (^∗^p < 0.05; Kruskal-Wallis test; six mice per genotype). (B) Electron micrographs show asymmetric synapses (red stars) in the CA1 stratum radiatum. The scale bar represents 0.5 μm. Quantification is shown on the right-hand side (^∗∗∗^p ≤ 0.001; ANOVA; five mice per genotype). (C) Representative mEPSC traces recorded at −60 mV from CA1 cells. (D) Quantification of mEPSC frequency and amplitude. Numbers inside bars indicate the number of cells recorded from at least seven mice per genotype (^∗^p < 0.05; Mann-Whitney test for frequency; Student’s t test for amplitude). Data are represented as mean ± SEM.

To determine the impact of Dkk1 expression on synapse stability, we measured excitatory synapses by the co-localization of pre- and postsynaptic markers (vGlut1 and PSD95, respectively) in the CA1 stratum radiatum. iDkk1 mice exhibited fewer excitatory synapses (∼40% decrease; Figure 3A). Consistently, we observed a similar decrease in the number of asymmetric (i.e., excitatory) synapses in the CA1 stratum radiatum by electron microscopy (Figure 3B). Thus, Dkk1 triggers the degeneration of glutamatergic synapses in the adult hippocampus. To evaluate neuronal connectivity, we recorded miniature excitatory postsynaptic currents (mEPSCs) using whole-cell patch-clamp recordings from CA1 neurons. Although no changes in mEPSC amplitude were observed, we found a significant decrease in mEPSC frequency (∼40%) in iDkk1 mice (Figures 3C and 3D), consistent with a decrease in excitatory synapse number.

**Figure 4:**
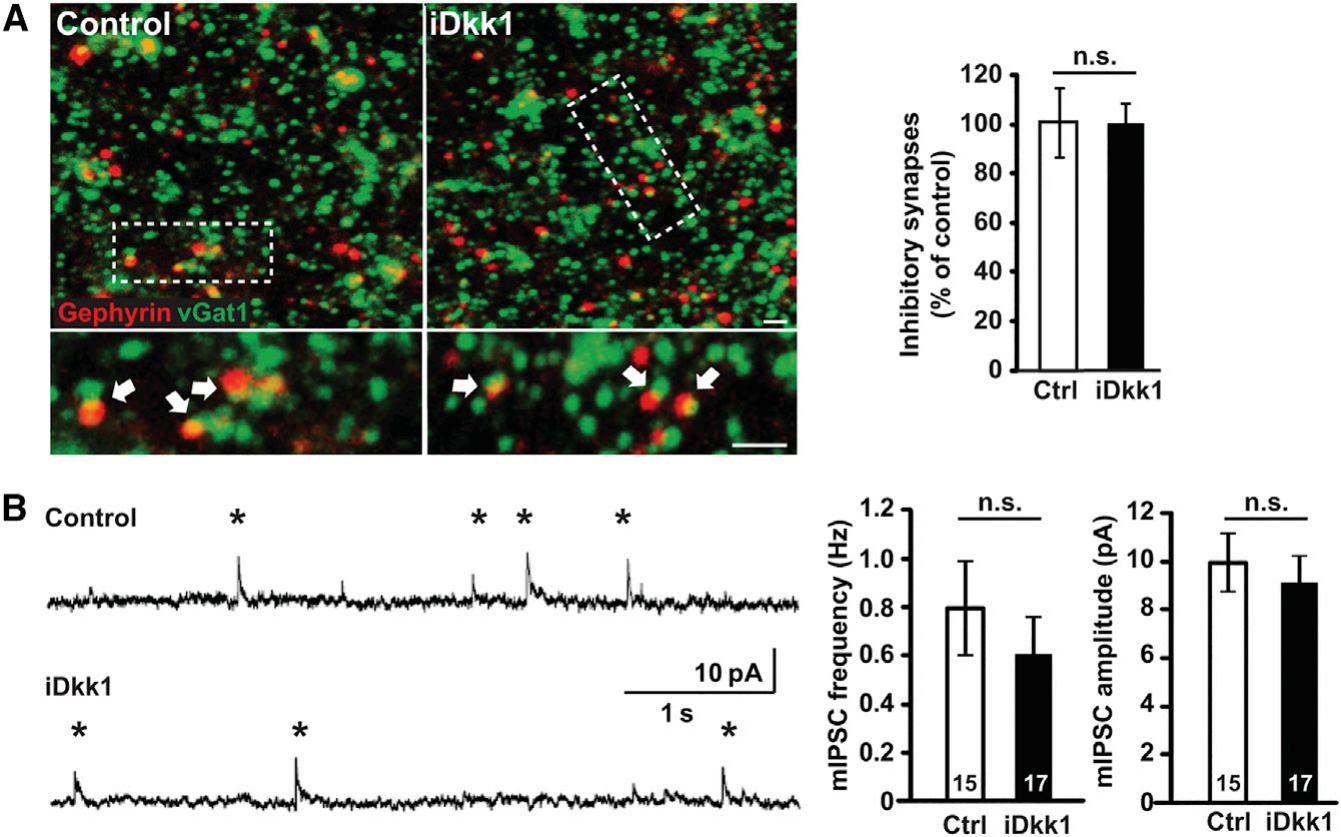
Dkk1 Does Not Affect Inhibitory Synapses in the Hippocampus in iDkk1 Mice (A) Confocal images of adult CA1 stratum radiatum show the presence of inhibitory synapses identified by co-localized presynaptic vGat and postsynaptic Gephyrin puncta (white arrows). The scale bars represent 2 μm. Quantification is shown on the side (Kruskal-Wallis test; three mice per genotype). (B) Representative mIPSC traces recorded at 0 mV from CA1 cells in acute hippocampal slices and quantification of mIPSC frequency and amplitude. Numbers inside bars indicate the number of cells recorded from at least seven mice per genotype (Mann-Whitney test for frequency; Student’s t test for amplitude). Data are represented as mean ± SEM.

In contrast, induced Dkk1 expression did not affect the number of inhibitory synapses in the CA1 region, as determined by co-localization of the pre- and postsynaptic markers vGat and gephyrin (Figure 4A). Consistently, the amplitude and frequency of miniature inhibitory postsynaptic currents (mIPSCs) in hippocampal CA1 neurons were unaffected by Dkk1 expression (Figure 4B). Thus, Dkk1 specifically affects the integrity of excitatory synapses without altering inhibitory synapses.

### Dkk1 Triggers Synaptic Disassembly by Blocking Canonical Wnt Signaling and Activating the RhoA-Rock Pathway

**Figure 5:**
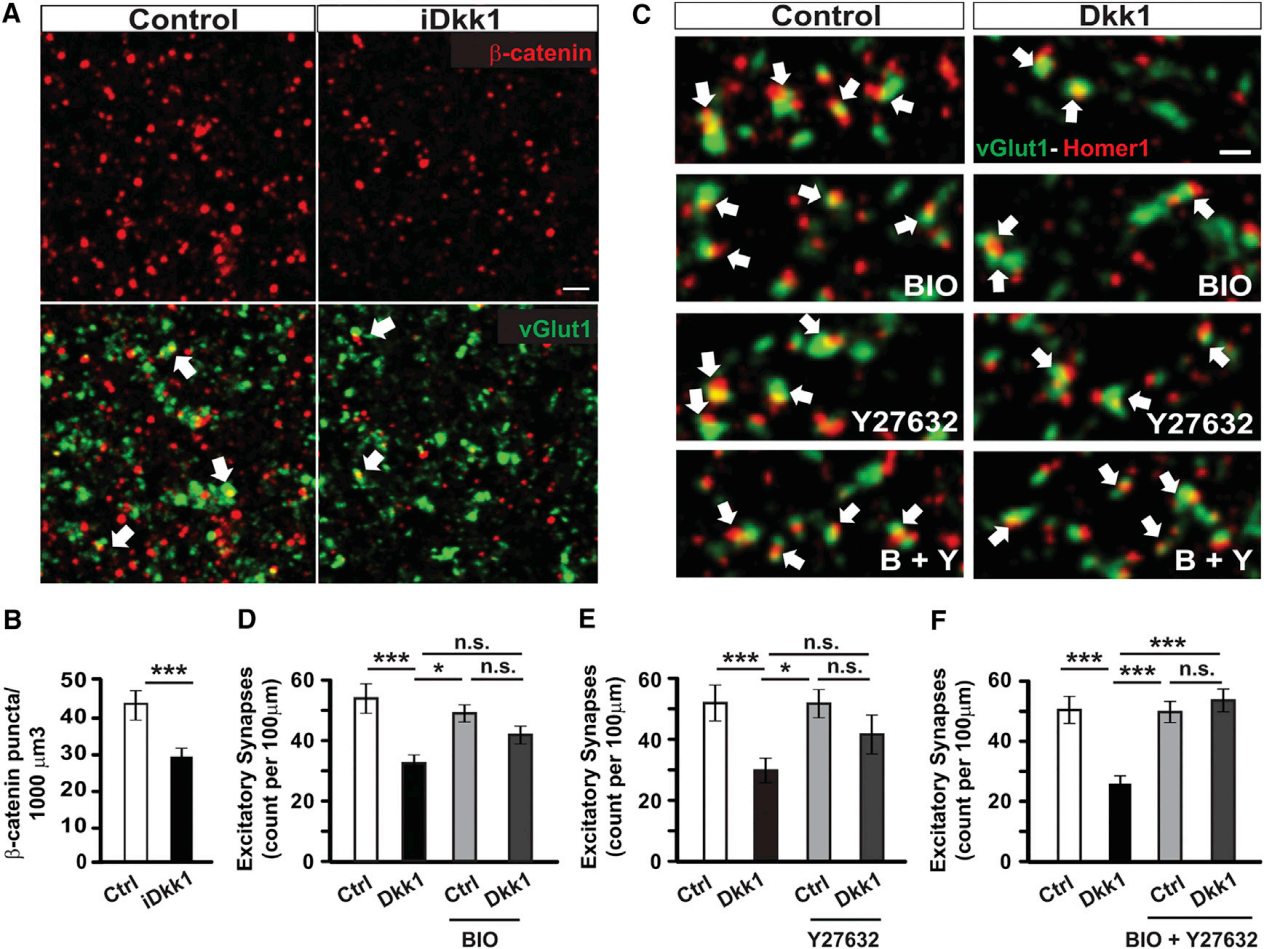
Dkk1 Induces Synapse Loss through Blockade of Canonical Wnt-Gsk3 Pathway and Activation of the RhoA-Rock Pathway (A) Distribution of β-catenin and vGlut1 puncta in the hippocampal CA1 stratum radiatum as indicated on the right corner. White arrows indicate that only few β-catenin puncta co-localize with vGlut1. The scale bar represents 2 μm (see also Figure S5). (B) Graph shows quantification of β-catenin puncta (^∗∗∗^p ≤ 0.001; ANOVA; four mice per genotype). (C) Confocal images show the presence of excitatory synapses (co-localized vGlut1 and Homer1 puncta; white arrows) in mature hippocampal neurons exposed to control or Dkk1 and specific Gsk3 and Rock inhibitors as indicated. The scale bar represents 2 μm. (D–F) Quantification of excitatory synapses per 100 μm dendrite after treatment with Dkk1 and with BIO, a Gsk3 inhibitor (D), with a Rock inhibitor, Y27632 (E), or with both BIO and Y27632 (F; ^∗^p < 0.05; one-way ANOVA test; n = 3 independent experiments per condition). Data are represented as mean ± SEM.

Dkk1 is a known specific Wnt antagonist that blocks canonical Wnt signaling [11, 12]. Wnt ligands bind to Frizzled (Fz) receptors and the co-receptors LRP6, resulting in the inhibition of Gsk3β-mediated phosphorylation and stabilization of β-catenin, which translocates to the nucleus and activates transcription [51] (Figure S5). In contrast, in the presence of Dkk1, binding of Wnts to Fz/LRP6 is blocked, resulting in enhanced Gsk3β-mediated β-catenin degradation by the proteasome pathway (Figure S5) [12, 51]. Thus, Dkk1 effectively blocks the function of several Wnts that signal through the LRP6 receptor. To investigate the impact of Dkk1 expression in canonical Wnt signaling, we evaluated β-catenin levels. Indeed, expression of Dkk1 resulted in fewer β-catenin puncta in the CA1 stratum radiatum of iDkk1 mice (Figures 5A and 5B), indicating that Dkk1 blocks the canonical Wnt-β-catenin pathway. Co-localization with the synaptic marker vGlut1 showed that most β-catenin puncta were extrasynaptic, indicating that the loss of β-catenin induced by Dkk1 was not due to synapse loss. These results suggest that Dkk1 expression blocks canonical Wnt signaling in the adult hippocampus.

Next, we evaluated whether Dkk1-mediated synaptic loss is due to blockade of canonical Wnt signaling. We used the specific Gsk3 inhibitor BIO (6-bromoindirubin-3′-oxime), which activates the Wnt pathway downstream of Dkk1 [52, 53]. Using a concentration of BIO, which does not affect synapse number on its own (Figures 5C and 5D), we found that this Gsk3 inhibitor partially occluded Dkk1-induced synapse disassembly (Figures 5C and 5D), suggesting that Dkk1 induces synapse loss through blockade of the Wnt-Gsk3β pathway but additional pathways might be involved. Dkk1 is mostly known as a specific and potent inhibitor of the Wnt-Gsk3β pathway; however, some studies have suggested that Dkk1 could activate non-canonical Wnt pathways [16, 54, 55, 56]. Although a role for the RhoA-Rock pathway in Dkk1 has not been reported in neurons, this cascade is of particular interest because it has been implicated in synaptic plasticity, learning, and memory and in Aβ-mediated synapse loss [57, 58, 59]. We therefore examined the role of this pathway in Dkk1-mediated synapse degeneration. Exposure to Y27632, a specific Rock inhibitor, partially prevented Dkk1-mediated synapse loss (Figures 5C and 5E). Given the partial protection by both Gsk3β inhibition and Rock inhibition on Dkk1-mediated synapse degeneration, we examined the combined effect of Gsk3β and Rock inhibitors and found complete blockade of Dkk1-induced synapse loss (Figures 5C and 5F). These results demonstrate a novel role for RhoA-Rock pathway in Dkk1 function and suggest that Dkk1 promotes synapse disassembly by blocking canonical Wnt signaling and activating the RhoA-Rock pathway.

### Synaptic Loss, Plasticity Defects, and Behavioral Impairment Are Reversible

**Figure 6:**
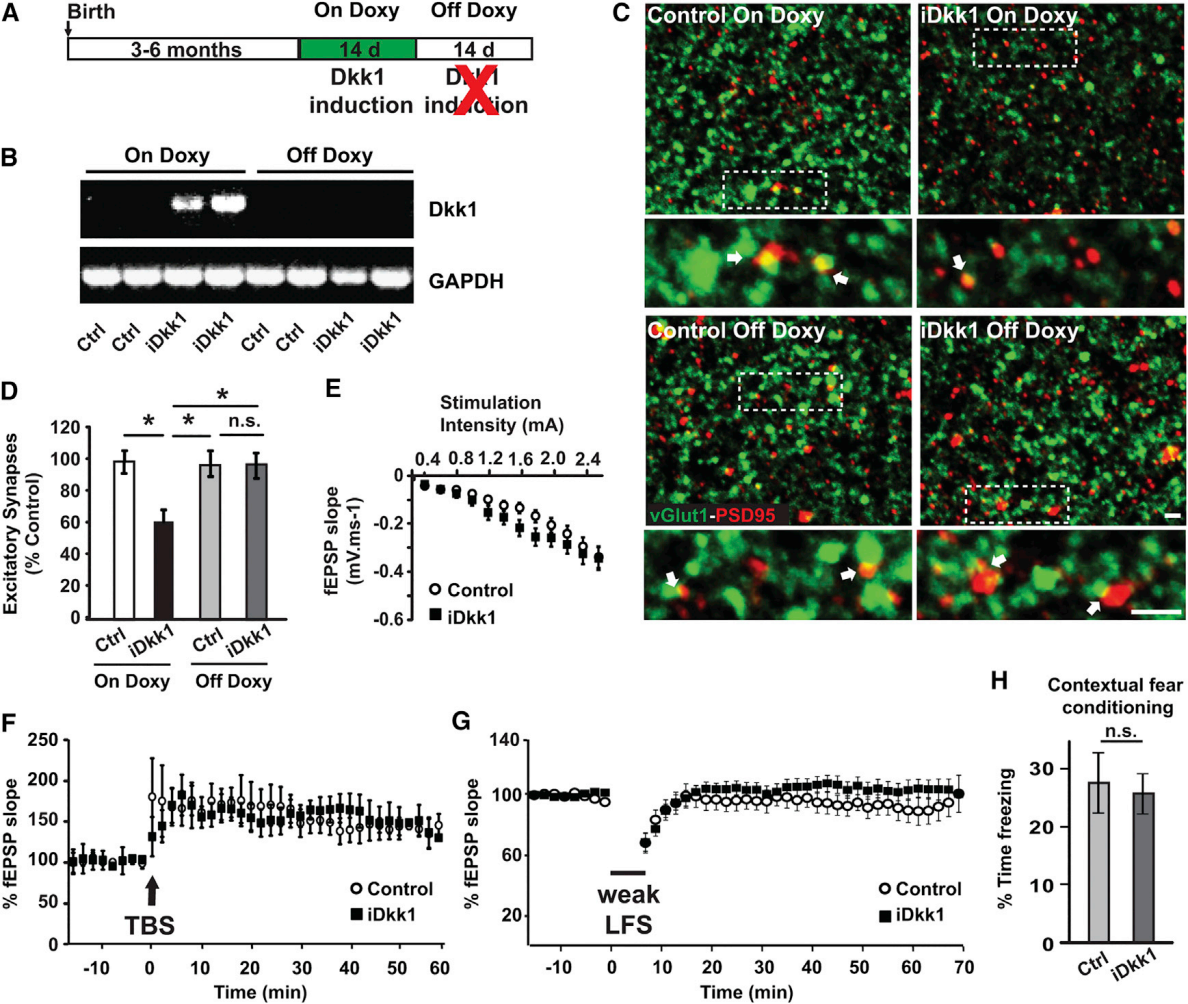
Synapse Loss, Long-Term Plasticity, and Memory Defects Are Reversible (A) Schematic representation of doxycycline feeding schedule in adult mice. (B) RT-PCR for Dkk1 in mice fed with doxycycline for 2 weeks (On Doxy) or after subsequent 2 weeks without doxycycline (Off Doxy). (C) Images of hippocampal CA1 show excitatory synapses (co-localized vGlut1 and PSD95 puncta; white arrows). The scale bar represents 2 μm. (D) Quantification of excitatory synapses (^∗^p < 0.05; Kruskal-Wallis test; six mice per genotype). (E) Input-output curves show no difference between control and iDkk1 mice after doxycycline withdrawal (nine slices from five controls and 11 slices from seven iDkk1 mice; repeated-measures ANOVA). (F) TBS-induced LTP in control and iDkk1 mice after doxycycline withdrawal (seven slices from six controls and nine slices from six iDkk1 mice; repeated-measures ANOVA). (G) Weak LFS failed to induce LTD at the SC-CA1 synapses of control or iDkk1 mice after doxycycline withdrawal (nine slices from seven controls and eight slices from five iDkk1 mice; repeated-measures ANOVA). (H) Percentage of freezing time evaluated 24 hr after the foot shock (15 control and 14 iDkk1 mice). Data are represented as mean ± SEM.

Diagnosis of neurodegenerative diseases is often made after substantial loss of synaptic connectivity has occurred. Thus, understanding the reversible nature of synaptic degeneration is crucial for developing therapies for the treatment of cognitive impairments in neurodegenerative diseases. We therefore examined whether Dkk1-mediated synapse loss and network dysfunction is reversible. We performed in vivo on-off experiments (Figure 6A), in which Dkk1 expression was induced for 2 weeks with doxycycline (On Doxy), followed by withdrawal of doxycycline for a further 2 weeks (Off Doxy). RT-PCR revealed that Dkk1 was expressed during the “on” period, but not after the “off” period, confirming that Dkk1 expression is tightly regulated by doxycycline (Figure 6B). Remarkably, the number of excitatory synapses fully recovered to control levels after doxycycline withdrawal (Figures 6C and 6D). These results demonstrate that, even after significant degeneration, the number of synaptic connections can be restored when Dkk1 expression is turned off in the adult hippocampus.

We then evaluated whether defects in basal transmission, long-term plasticity, and long-term memory could be reversed in iDkk1 mice. We found that cessation of Dkk1 expression resulted in full recovery of basal synaptic transmission as indicated by the overlapping input-output curves from control and iDkk1 mice (Figure 6E). Notably, TBS fully induced LTP in iDkk1 mice after termination of Dkk1 expression (Figure 6F). Moreover, weak LFS induced short-term depression without inducing LTD in both control and iDkk1 mice (Figure 6G). Finally, using the contextual fear-conditioning test, we found that turning off Dkk1 expression in iDkk1 mice completely recovers their ability to form long-term memory, as the percentage of freezing time was similar to control mice (Figure 6H). Taken together, these studies show the remarkable capacity of the adult hippocampus to regenerate synapses that integrate into functional neuronal circuits. They also demonstrate that synapse degeneration can be reversed in the adult mouse brain by modulating Wnt signaling.

## Discussion

Here, we report that deficiency in Wnt signaling by inducibly expressing the specific Wnt antagonist Dkk1 in the adult hippocampus triggers the loss of excitatory synapses in CA1 neurons, impairs synaptic plasticity, and alters hippocampal-dependent function. These defects occur in the absence of cell death and require the combined activation of Gsk3β and Rock. Notably, Dkk1-induced synaptic defects are fully reversed upon cessation of Dkk1 expression. Our findings demonstrate that iDkk1 mice provide a unique model system to study the in vivo impact of deficient Wnt signaling on synapse vulnerability and to elucidate the molecular mechanisms that contribute to synapse regeneration after substantial synapse loss and dysfunction.

In the adult hippocampus, Dkk1 expression blocks Wnt signaling without affecting cell viability or the stem cell niche. Previous studies have shown that Dkk1 can promote cell death in models of AD, epilepsy, and ischemia [13, 29, 60, 61] and affect adult neurogenesis by modulating the generation of immature neurons in the adult DG [30]. However, we found no evidence of increased cell death or an effect on the number of newborn neurons in the adult hippocampus of iDkk1 mice. This could be attributed to low levels of Dkk1 expression after 2 weeks induction of this protein. Given the direct effect of Wnts on synapses [62, 63, 64], our results suggest that Dkk1 induces synaptic vulnerability by directly targeting synapses.

Blockade of Wnt signaling with Dkk1 specifically affects excitatory synapses in the adult hippocampus, resulting in decreased mEPSC frequency and reduced excitatory synaptic transmission. In contrast, Dkk1 does not affect the number of inhibitory synapses or mIPSC frequency and amplitude. In the adult striatum, Dkk1 also induces the loss of excitatory synapses [22]. Together, these results highlight the crucial role for Wnt signaling in the maintenance of functional excitatory synapses in the adult brain. Although the mechanism by which Dkk1 specifically affects excitatory, but not inhibitory, synapses remains unknown, recent studies showed that LRP6 is predominantly present at excitatory synapses [65] and that deficiency in LRP6 affects excitatory synapses in the hippocampus [10]. These results suggest that Dkk1 acts through LRP6, which is upstream of the Wnt-Gsk3β pathway. Consistent with the inhibition of this pathway, the number of β-catenin puncta decreases in hippocampal CA1 following Dkk1 expression. Thus, induced expression of Dkk1 compromises the canonical Wnt pathway.

Dkk1 induces synapse degeneration by modulating the Wnt-Gsk3β and the Rock pathways. Our studies reveal that inhibiting Gsk3β with BIO partially blocks Dkk1-mediated synapse disassembly, suggesting that additional pathways might be involved. Previous studies showed that activation of the RhoA-Rock pathway leads to spine loss and mediates Aβ-induced synapse loss [57, 58, 59]. Interestingly, we found that Rock inhibition partially blocks Dkk1-induced synapse degeneration. In contrast, inhibition of both Gsk3β and Rock completely protects synapses against Dkk1. Therefore, we have identified Rock as a novel downstream target for Dkk1. How Dkk1 activates Gsk3β and Rock pathways is unknown. Both signaling cascades could influence the stability of the synapse by modulating different targets, such as β-catenin and microtubules in the case of Gsk3β or the actin cytoskeleton through the Rock pathway. Alternatively, both pathways could interact as recently reported for the role of Wnts in cell migration [66]. Future studies will elucidate the downstream events by which these two pathways contribute to Dkk1-mediated synapse vulnerability.

Induced Dkk1 expression affects long-term plasticity and memory. iDkk1 mice exhibit impaired hippocampus-dependent function as demonstrated by defects in contextual fear memory and spatial learning and memory. These results are in agreement with a previous study suggesting a role for Wnt signaling in memory [16, 67]. Memory deficits have been associated with defects in long-term plasticity in the hippocampus [68, 69, 70]. Consistently, iDkk1 mice exhibit a significant impairment in LTP, a defect that could be due to the loss of 40% of excitatory synapses [47] and/or to the impaired ability of remaining synapses to respond to LTP induction. We also demonstrate a novel function for Wnt signaling in LTD. Previous studies showed that Gsk3β activation suppresses LTP [71] and enhances LTD [72], suggesting a role for Gsk3β downstream of Dkk1-mediated synaptic dysfunction.

Understanding the molecular pathways that promote the regeneration of synapses that integrate into networks is crucial for developing effective therapies to promote functional recovery. Here, we report that synapse loss, defects in synaptic plasticity, and memory deficits can be fully restored in iDkk1 mice after cessation of Dkk1 expression. Our findings demonstrate the remarkable capacity of adult neurons to regenerate functional circuits after substantial synapse loss and highlights that Wnt signaling is a targetable pathway in neurodegenerative diseases.

## Experimental Procedures

### Animals

Experiments were performed according to the Animals Scientific procedures Act UK (1986). Double transgenic mice (iDkk1) were obtained as described in [22]. Adult (3–6 months old) iDkk1 and control mice (tetO-Dkk1, CaMKIIα-rtTA2, or wild-type littermates) were fed with pellets containing 6 mg/kg doxycycline (Datesand Group) ad libitum for 2 weeks, unless otherwise indicated. For the on-off experiment, 2 weeks of doxycycline feeding was followed by 2 weeks of feeding with the original diet. Males were used for electrophysiological and behavioral experiments, whereas both genders were used for cellular biology experiments. See the Supplemental Experimental Procedures for more details.

### Hippocampal Culture, Cell Transfection, and Drug Treatment

Hippocampal cultures were prepared from embryonic day 18 (E18) embryos of Sprague-Dawley rats as described previously [73] and maintained for 21 days in vitro (DIVs). Purified recombinant Dkk1 (200 ng/mL; PeproTech) was applied to cells for 2 hr in the presence or absence of the Gsk3 inhibitor BIO (200 nM; BioVision Technologies) and ROCK inhibitor Y27632 (10 μM; Selleckchem). See the Supplemental Experimental Procedures for further details.

### Immunofluorescence Staining

Brain slices from control and iDkk1 mice were incubated in blocking solution (10% donkey serum and 0.02% v/v Triton X-100 in PBS) for 4 hr at room temperature (RT). Primary antibodies were incubated overnight at 4°C. Secondary antibodies conjugated with Alexa 488, 568, or 647 (1:600; Invitrogen) were incubated at RT for 2 hr. In some experiments, brain sections were incubated with Hoescht for 5 min. Samples were washed in PBS and mounted in Fluoromount-G (SouthernBiotech).

Hippocampal neurons were fixed in 4% paraformaldehyde (PFA) in PBS for 20 min at RT, permeabilized for 5 min in 0.05% v/v Triton X-100 in PBS, and blocked in 5% BSA for 1 hr. Primary antibodies and secondary antibodies were each incubated for 1 hr at RT. Samples were washed in PBS and mounted in FluorSave Reagent (Millipore). See the Supplemental Experimental Procedures for more details.

### Image Acquisition and Analyses

For evaluation of synaptic puncta, stacks of eight equidistant planes (0.2 μm; 76 × 76 nm/pixel) from hippocampal slices and cultured neurons were acquired on an Olympus FV1000 confocal microscope using a 60 × 1.35 numerical aperture (NA) oil objective. Four to seven fields were taken per brain slice, and three to four slices were analyzed per mouse. For hippocampal neurons, eight to ten image stacks of EGFP-transfected cells were taken per condition. Analysis was performed in Volocity software (PerkinElmer). See the Supplemental Experimental Procedures for more details.

### Electrophysiology

For field potential recordings, parallel bipolar stimulation electrodes were placed in the stratum radiatum of the CA1 region and Schaeffer collateral fibers were stimulated with 0.1 ms duration constant-current paired-pulses (pulse interval 50 ms) delivered to the pathway at intervals of 10 s. Stimulus current was adjusted at the beginning of each recording to give a response approximately 50% of the maximum fEPSPs slope, after recording an input-output curve. fEPSPs were monitored using low-resistance glass pipettes (1–2 MΩ), filled with 4 mM NaCl in ACSF. Slices were subjected to a 15–20 min period of pre-LTP/pre-LTD baseline measurement every 10 s. Provided that the control response did not change by more than 5% during this 15–20 min period, LTP or LTD was induced. LTP was induced by a TBS protocol, which involved delivering two TBSs at an interval of 10 s, and each TBS was composed of five trains of stimuli at intervals of 200 ms, where each train contained four stimuli at 100 Hz. Two protocols of LFS consisting of two trains of 900 pulses delivered at 2 Hz with a 2.5 min gap (strong LFS) or one train of 900 pulses delivered at 2 Hz (weak LFS) were used to induce a LTD. Stimulus intensity for the TBS and LFS was the same as baseline recordings. Paired-pulse fEPSPs (20 Hz) were recorded at intervals of 10 s for at least 50 min after delivery of the TBS or LFS, and the slope of each fEPSP was measured. fEPSP-PPR was calculated as the ratio of the slope of the second to the first fEPSP. Recordings were made using an Axopatch 200B amplifier, filtered (1 kHz) and digitized (10 kHz), and then analyzed using WinEDR software or WinWCP software (freely available at http://spider.science.strath.ac.uk/sipbs/software_ses.htm). For these experiments and patch-clamp recordings, see the Supplemental Experimental Procedures for further information.

### Behavioral Studies

For all behavioral tests, adult male mice were handled daily for approximately 2 min, at least 4 days before the beginning of the test. Throughout experimentation and data analysis, the experimenter was blind to genotype. MWM, contextual fear conditioning, T-maze spontaneous alternation, open field, and elevated plus maze tasks are described in the Supplemental Experimental Procedures.

### Statistical Analyses

For behavioral analyses, each mouse group consisted of at least seven animals. For immunofluorescence, data were generated from three or more independent experiments, each with one to four mice per genotype. All results were expressed as mean ± SEM. Statistical significance was calculated on the basis of a Student’s t test, one-way ANOVA, or ANOVA for repeated measures when samples were normally distributed, followed by Scheffe or Bonferroni posteriori comparisons. Mann-Whitney or Kruskal-Wallis tests were used for non-normally distributed data followed by Dunn-Sidak posteriori comparisons (^∗^p < 0.05, ^∗∗∗^p ≤ 0.001, ^∗∗^p ≤ 0.01).

## Author Contributions

P.C.S. conceived the project. All authors contributed to the design of experiments, interpretation of the data, and writing of the manuscript. S.P. performed the initial characterization of the Dkk1 induction in the adult hippocampus. D.L., A.M., and M.P. performed behavioral experiments; D.L., S.G., A.M., and F.M. performed cell biology experiments; and A.M. and M.S.-R. performed the electrophysiological recordings. F.C. contributed to design and analysis of behavioral assays. P.C.S. and A.G. provided funding and supervised the project.

## Supplemental Information

Document S1. Supplemental Experimental Procedures and Figures S1–S5

Document S2. Article plus Supplemental Information
