# Supplementary material for: Reversal of Synapse Degeneration by Restoring Wnt Signaling in the Adult Hippocampus
Source: Curr Biol. 2016 Oct 10;26(19):2551–61. doi: 10.1016/j.cub.2016.07.024 (PMC5070786; doi:10.1016/j.cub.2016.07.024)
Supplement: Document S1. Supplemental Experimental Procedures and Figures S1–S5 [file mmc1.pdf]

Current Biology, Volume *26*

Supplemental Information

Reversal of Synapse Degeneration by Restoring Wnt Signaling in the Adult Hippocampus

Aude Marzo, Soledad Galli, Douglas Lopes, Faye McLeod, Marina Podpolny, Margarita Segovia-Roldan, Lorenza Ciani, Silvia Purro, Francesca Cacucci, Alasdair Gibb, and Patricia C. Salinas

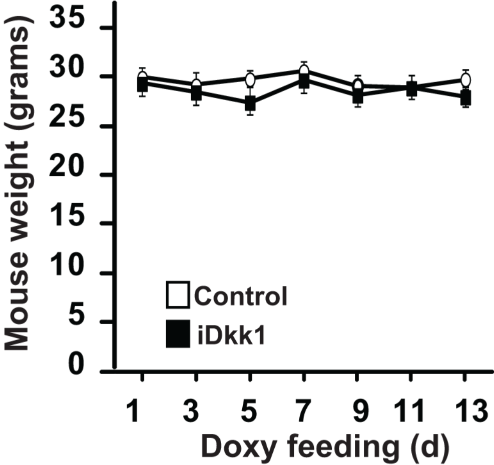

Mouse weight (grams)
35
30
25
20
15
10
5
0
Control
iDkk1
1
3
5
7
9
11
13
Doxy feeding (d)

Figure S1

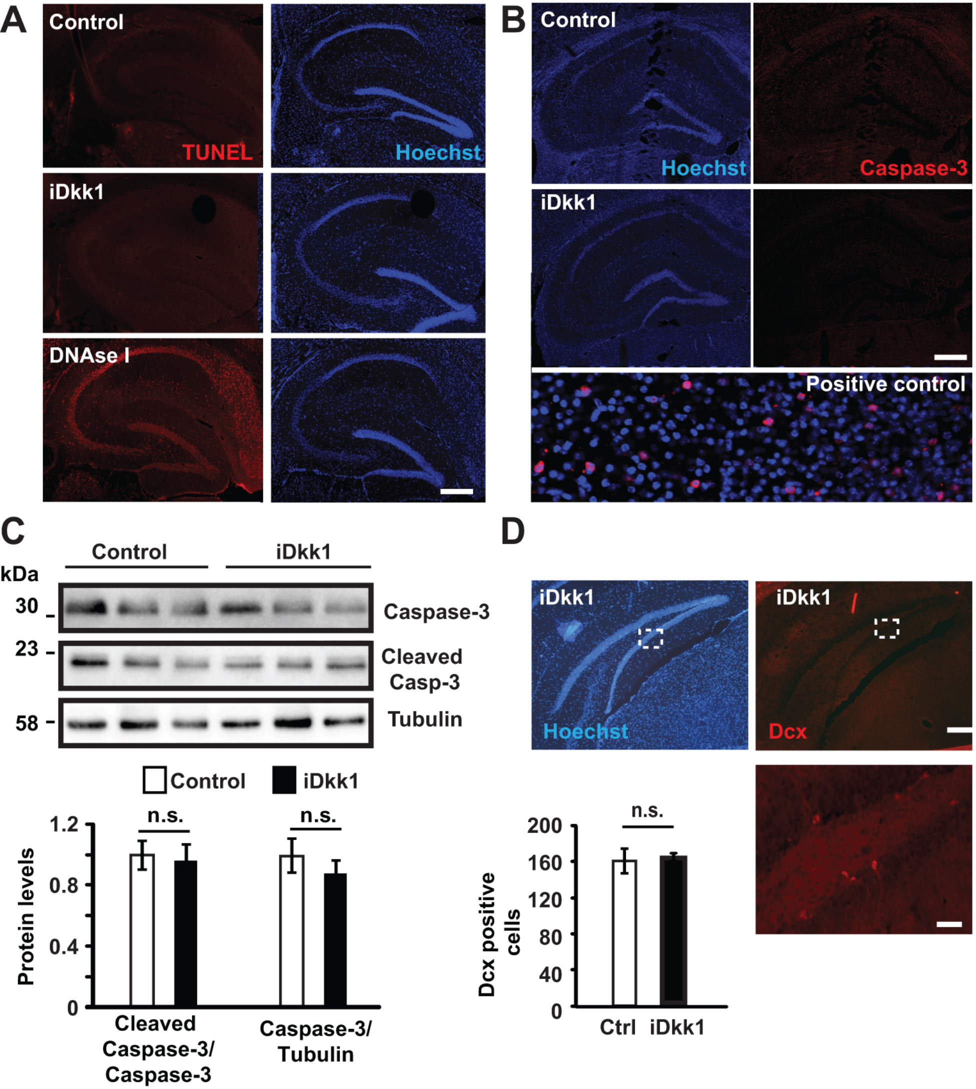

A
Control
TUNEL
Hoechst
iDkk1
DNAse I
B
Control
Hoechst
Caspase-3
iDkk1
Positive control
C
Control
iDkk1
kDa
30
23
58
Caspase-3
Cleaved Casp-3
Tubulin
Control
iDkk1
n.s.
n.s.
1.2
0.8
0.4
0
Protein levels
Cleaved Caspase-3/ Caspase-3
Caspase-3/ Tubulin
D
iDkk1
Hoechst
iDkk1
Dcx
n.s.
200
160
120
80
40
0
Dcx positive cells
Ctrl
iDkk1

Figure S2

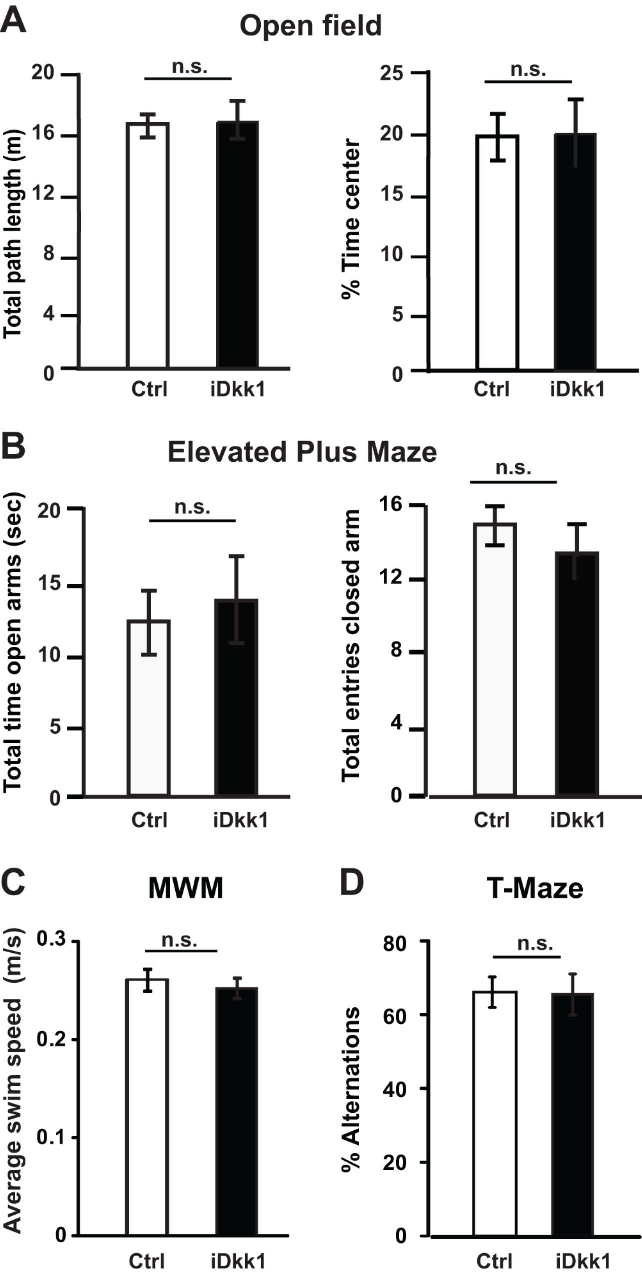
A
Open field
n.s.
Total path length (m)
20
16
12
8
4
0
Ctrl
iDkk1
n.s.
% Time center
25
20
15
10
5
0
Ctrl
iDkk1
B
Elevated Plus Maze
n.s.
Total time open arms (sec)
20
15
10
5
0
Ctrl
iDkk1
n.s.
Total entries closed arm
16
12
8
4
0
Ctrl
iDkk1
C
MWM
n.s.
Average swim speed (m/s)
0.3
0.2
0.1
0
Ctrl
iDkk1
D
T-Maze
n.s.
% Alternations
80
60
40
20
0
Ctrl
iDkk1

Figure S3

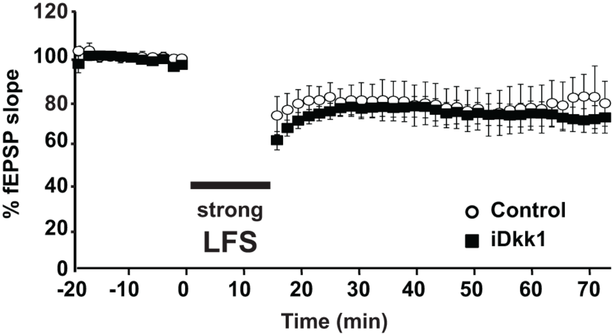
120
100
80
60
40
20
0
% fEPSP slope
-20
-10
0
10
20
30
40
50
60
70
Time (min)
strong
LFS
Control
iDkk1

Figure S4

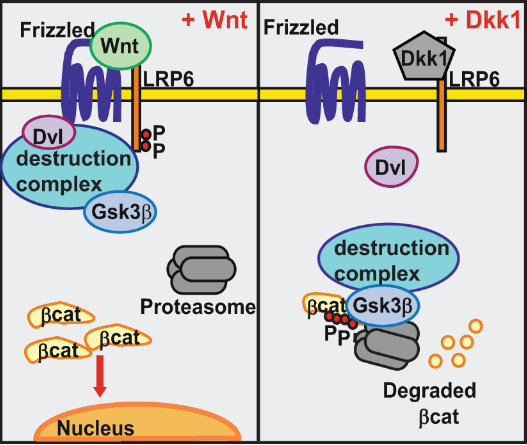
Frizzled
Wnt
+ Wnt
LRP6
P
P
Dvl
destruction complex
Gsk3β
Proteasome
βcat
βcat
βcat
Nucleus
Frizzled
+ Dkk1
Dkk1
LRP6
Dvl
destruction complex
βcat
Gsk3β
P
P
Degraded βcat

Figure S5

Supplemental Legends

Figure S1, refers to Figure 1A: Dkk1 does affect food intake. Weight of control and iDkk1 mice monitored during doxycycline administration. Data are mean ± SEM.

Figure S2, refers to Figure 1D: Dkk1 does not affect overall hippocampus morphology, cell viability and adult neurogenesis in iDkk1 mice. (A) TUNEL assay shows no difference in cell death. DNAse I was used as a positive control. Scale bar: 250 μm. (B) Cleaved caspase 3 staining reveals the level of cell death in the hippocampus. Scale bar: 250 μm. Positive control corresponds to cortical slices from P2 mice. (C) Western blot from hippocampus lysates shows no changes in the levels of caspase-3 activation in iDkk1 mice. Graph demonstrates that levels of cleaved caspase-3 in relation to uncleaved caspase-3 are equal in both groups. Uncleaved caspase-3 levels are unaffected in iDkk1 mice. (Student's *t*-test, 3 animals per genotype). (D) Images and quantification of newborn neurons labelled with Doublecortin (Dcx) in the dentate gyrus from iDkk1 mice (Student's *t*-test, 4 mice per genotype). Scale bar: 100 μm in panoramic view and 50 μm in high magnification images. Data are presented as mean ± SEM.

Figure S3, refers to Figure 1: Induced expression of Dkk1 does not affect anxiety-like behavior in iDkk1 mice. (A) Total path length in the Open Field (left panel) and percentage of time spent in the center of the open field arena (right panel; Student's *t*-test). (B) Time spent in the open arms of the elevated plus maze (left panel) and total number of entries to the closed arms of the elevated plus maze (right panel; Student's *t*-test, 15 control and 11 iDkk1 mice). (C) Average swimming speed in the Morris Water Maze (MWM, Student's *t*-test). (D) Percentage of alternation in the T-maze test (15 control and 10 iDkk1 mice, *$p<0.05$, Student's *t*-test). Results are shown as mean ± SEM.

Figure S4, refers to Figure 2: Dkk1 expression does not alter LTD induced by a strong LFS. LTD induced by delivery of a strong LFS, starting at time zero (8 slices from 7 control (open circles) and 14 slices from 9 iDkk1 mice (black squares); repeated measures ANOVA). Data are presented as mean ± SEM.

Figure S5, refers to Figure 6: Illustration of the Wnt canonical pathway. Binding of Wnt to Frizzled (Fz) and LRP6 receptors recruits Dishevelled (Dvl) resulting in inhibition of Gsk3β-mediated phosphorylation of β-catenin (β-cat) promoting its stabilization (left panel). In contrast, Dkk1 binding to LRP6 (right panel) prevents the formation of the Wnt/Fz/LRP6 complex, thus inhibiting the canonical Wnt pathway resulting in the activation of Gsk3β and in the degradation of β-catenin by the proteasome.

Supplemental Experimental Procedures

Animals

Double transgenic mice (iDkk1) were obtained by crossing tetO-Dkk1 transgenic mice [S1] with CaMKIIα-rtTA2 transgenic mice [S2]. TetO-Dkk1 transgenic mice and CaMKIIα-rtTA2 were crossed in heterozygous state. Both mouse lines were bred in a C57BL/6J background for at least 6 generations. Genotyping was performed using DNA from ear biopsies, using the following primers: CaMKIIα-rtTA: forward 5' TGCCTTTCTCTCCACAGGTGTCC 3' and reverse 5' GAGAGCACAGCGGAATGAC 3'; tetO-Dkk1: forward 5' GCGTCCTTCGGAGATGATGG 3', and reverse 5' AAATGGCTGTGGTCAGAGGG 3'.

Hippocampal culture, cell transfection and drug treatment

High-density hippocampal cultures (250 cells/mm^2) were transfected at 7-8 DIV using calcium phosphate with EGFP-actin to visualize dendrites and spines.

RT-PCR analyses

RNA was extracted from the hippocampus of 3 adult mice (3 months old) using Trizol (Invitrogen) and treated with DNAse I (Sigma). First, strand complementary DNA synthesis was performed with AMV Reverse Transcriptase (Promega) according to the manufacturer's instruction. PCR was performed using GoTaq Polymerase (Promega). The primers used are forward 5' ATTCCAACGCGATCAAGAAC 3' and reverse 5' GCTTGGTGCATACCTGACCT 3'.

In situ hybridization

In situ hybridization was performed as previously reported [S3]. Brains were snap-frozen in pre-cooled isopentane and kept at –80°C. Sagittal sections (12 μm), cut in a cryostat, were air-dried and fixed in 4% paraformaldehyde (PFA) in PBS for 20 min. Slides were incubated with anti-sense or sense Dkk1 cRNA probe [S4] prepared using the DIG-RNA labelling kit (Invitrogen), overnight at 55° C. Slides were then washed and incubated with goat anti-DIG-AP for 3.5 hours. Following washes, samples were incubated with nitro-blue

tetrazolium (NBT) and 5-bromo-4-chloro-3'-indolyphosphate (BCIP) for chromogenic detection, washed in TE buffer to stop the reaction and mounted in Fluoromount-G (SouthernBiotech).

Preparation of brain sections

Brains from control and iDkk1 mice were rapidly dissected and placed in artificial cerebrospinal fluid (ACSF) containing in mM: 126 NaCl, 3 KCl, 2 $CaCl_2$, 1 $MgCl_2$, 1.25 NaH_2PO_4, 26 $NaHCO_3$, 10 D-glucose, pH 7.4. For cleaved caspase 3, NeuN and Doublecortin staining, brains from perfused mice were post-fixed overnight (4% PFA), immerged in 30% sucrose/PBS and frozen in pre-cooled isopentane. Sections (30 µm) were collected onto Superfrost Plus–VWR slides and stored at -20 °C.

Immunofluorescence staining

List of primary antibodies used: vGlut1 (1:2000; Millipore), PSD95 (1:500; Thermo Scientific), Homer1 (1:1000; Synaptic Systems), vGat (1:500; Synaptic Systems), Gephyrin (1:500; Synaptic Systems), β-catenin (1:500; BD transduction labs), cleaved caspase 3 (1:500, Cell Signaling), NeuN (1:500, Millipore), Doublecortin (1:200; Santa Cruz) and GFP (1:1000; Millipore)

TUNEL staining was performed with ApoptoTag® Red *In Situ* Apoptosis detection kit (Chemicon International). For positive controls, brain slices were treated with 1 µg/mL DNAse I at RT**.** For cleaved caspase-3 experiment, positive control consists on horizontal acute brain slices of cortex from P2 mice were incubated at 37 ° C for a period of 4 h and then stained as above.

Image acquisition and analyses

For NeuN experiments, image stacks of 8 equidistant planes were captured on an Olympus FV1000 confocal microscope using a 20× 1.35 NA oil objective. Four to seven fields of the CA1 pyramidal cell layer were acquired per animal. Positive labelled neurons were manually counted in Volocity software (Perkin Elmer) using maximum projection stacks. For Doublecortin experiments, single plane images were captured on an Olympus BX60 microscope with a 10× 1.3 NA objective. Doublecortin positive cells were counted and analysed as previously described [S5].

Analyses were performed in Volocity software. Pre- and postsynaptic puncta number was determined using customized Volocity protocols based on standard thresholding techniques. For hippocampal cells, three dendrites were cropped from maximum projection images of each cell and synaptic puncta were visualized using threshold protocols and normalized to the length of the dendrite. Synapses were defined as the co-localization between vGlut1 and Homer1 puncta.

Electron microscopy

Samples were prepared as described previously [S6]. Brains were fixed with 4% PFA, 0.5% glutaraldehyde in 0.1 M Milloning phosphate buffer, pH 7.4 overnight at 4°C. Coronal sections (200 µm) were cut on a vibratome and post fixed in 1% osmium tetroxide in cacodylate buffer for 1 h, stained in aqueous uranyl acetate for 45 min, dehydrated in graded alcohol and embedded in resin. Ultra-thin sections (70 nm) of silver-gold interference colour were cut and collected on a 200 mesh-grid. Photographs were taken at 40,000X magnification with a JEOL 1010 microscope. Asymmetric synapses were considered when a clear pre- and postsynaptic membrane was visualized, with the presence of a prominent PSD and vesicles in the pre-synaptic terminal [S7, 8].

Western Blot

Homogenates from hippocampus of iDkk1 and control animals (3-4 mice each) were run on 10% SDS-PAGE gels, and Western blots were probed with antibodies against total caspase-3 (1:1000, Synaptic Systems), cleaved caspase 3 (1:1000, Synaptic Systems) and α-tubulin (1: 2000, Sigma). Measurements of band intensity were performed using ImageJ software (NIH).

Electrophysiology

Brains were quickly dissected and transversal hippocampal slices were collected in high-sucrose-ACSF containing in mM: NaCl 75, $NaHCO_3$ 25, KCl 2.5, $NaHPO_4$ 1.25, kynurenic acid 1.25, pyruvic acid 2, EDTA 0.1, $CaCl_2$ 1, $MgCl_2$ 4, D-glucose 25 and sucrose 100, bubbled with 95% O_2/5% CO_2. Slices were kept at RT for patch clamp recordings or placed at 34°C for 1-2h before recordings for field potential recordings.

For field potential recordings, brain slices (400 µm thick) were placed in a chamber continuously perfused at 30°C with recording solution containing in mM: NaCl 125, $NaHCO_3$ 25, KCl 2.5, NaH_2PO_4 1.25, $CaCl_2$ 2, $MgCl_2$ 1 and D-glucose 25 and bubbled with 95% O_2/5% CO_2.

For patch clamp recordings, brain slices (300 µm thick) were placed in a chamber on an upright microscope and continuously superfused at room temperature with ACSF recording solution (in mM: NaCl 125, $NaHCO_3$ 25, KCl 2.5, $NaHPO_4$ 1.25, $CaCl_2$ 1, and D-glucose 25), bubbled with 95% O_2/5% CO_2. CA1 hippocampal cells were patched in whole cell voltage-clamp configuration using pipettes (resistance 5–8 MΩ) pulled from

borosilicate glass (Harvard GC150F-7.5) and filled with pipette solution containing in mM: D-gluconic acid lactone 139, HEPES 10, EGTA 10, NaCl 10, $CaCl_2$ 0.5, $MgCl_2$ 1, ATP 1 and GTP 1, adjusted to pH 7.2 with CsOH. When recording miniature currents, 100 nM TTX was included in the recording solution. Miniature or evoked EPSCs were recorded at -60 mV in the presence of 10 µM bicuculline and 50 µM AP-5, whereas IPSCs were recorded at 0 mV in the presence of 50 µM AP-5.

Behavioral studies

Morris Water Maze

Control and iDkk1 mice were fed with doxycycline for 5 days before the beginning of the task, and doxycycline feeding was maintained throughout the test. The test was performed in a circular 120 cm diameter pool as previously described [S9] with a few modifications. Briefly, the paradigm consisted of two training phases: 4 days with a visible platform, followed by 5 days (acquisition phase) with a hidden platform. For each training phase, a four trials/day training protocol with 10 min inter-trial intervals and maximum trial duration of 90 sec was used. In the first phase, mice were trained to locate a pseudo-randomly placed platform with a local cue (flag) protruding out of the water surface. At this stage, curtains were drawn around the pool to occlude any distal cues. During the second training phase, mice were trained to find a hidden platform with the extra-maze cues visible around the room. During probe trials platform was removed from the pool and mice were allowed to locate the platform for up to 90 sec. Probe trials were conducted before the fourth day of training (Probe I) and 24 h after the last day of training (Probe II). All trials were recorded with a camera located in the ceiling and images were analyzed using the DacqTrack-WaterMaze tracking system (Axona Ltd.).

Contextual Fear Conditioning

Mice were placed into a conditioning chamber (Med Associates Inc.) located in a soundproof box, equipped with a video camera. The conditioning chamber floor was a stainless steel grid used for shock delivery. A speaker mounted on the wall was used to deliver the tone. The contextual fear conditioning protocol used was as previously described [S10]. Briefly, on the conditioning day, mice were allowed to acclimatize to the room. Mice were then placed individually in the conditioning chamber and after a 120-sec introductory period, a tone (80 dB, 3.0 kHz) was presented for 30 sec, the last 2 sec of which coincided with a foot-shock (0.7 mA). Contextual fear memory was tested 24 h after training by re-exposure to the conditioning chamber for 5 min, with no shock delivery. Freezing behavior (defined as complete lack of movement, except for respiration) was scored for 2 sec every 5 sec by an experimenter blind to the genotype of the mice. Baseline freezing was considered as the levels of freezing prior to the presentation of the stimulus.

T-maze spontaneous alternation

The enclosed T-maze consisted of 2 arms of 27 x 7 x 10 cm each, made of white Formica. A partition extended 7 cm from the back of the T into the start arm. A set of three guillotine doors was used to separate the entrance of each arm. At the beginning of the test all the guillotine doors were raised, except for the one located at the end on the starting arm. Each mouse received eight consecutive trials in the enclosed T-maze. Each trial consisted in putting individually the animal at the entrance of the main arm and allowing the mouse to freely run to the goal arm. After the mouse has entered the goal arm, the guillotine door was closed and the animal was confined in the chosen goal arm for 30 sec. The mouse was then returned to the start partition, where it was held for 30 sec. Results are expressed as the number of correct alternations over the total number of possible correct alternations. Correct alternation was scored whenever the mouse entered into a previously unvisited arm.

Open Field

The apparatus consisted of a 45 x 45 x 45 cm wooden box open at the top, illuminated with a dim light. Mice were placed individually in the center of the arena, in a position equidistant to the walls, and allowed to explore the open field for 300 seconds. Mice were tracked using a video camera fixed to the ceiling of the room and connected to a digital video tracking system (HVS Image Ltd.). Distance travelled and time spent in the central and peripheral areas were monitored by the DacqTrack tracking system (Axona Ltd.).

Plus-maze

The apparatus, made of wood laminated with white formica, consisted of four 30 x 5 cm arms. Two of the arms were surrounded by 15 cm height walls. The apparatus was elevated 40 cm above the floor and lit by dim light. Mice were placed individually in the central area (neutral area) and monitored for 300 sec as described above.

Supplemental References

S1. Chu, E.Y., Hens, J., Andl, T., Kairo, A., Yamaguchi, T.P., Brisken, C., Glick, A., Wysolmerski, J.J., and Millar, S.E. (2004). Canonical WNT signaling promotes mammary placode development and is essential for initiation of mammary gland morphogenesis. Development *131*, 4819-4829.

S2. Michalon, A., Koshibu, K., Baumgartel, K., Spirig, D.H., and Mansuy, I.M. (2005). Inducible and neuron-specific gene expression in the adult mouse brain with the rtTA2S-M2 system. Genesis *43*, 205-212.

S3. Pasterkamp, R.J., De Winter, F., Holtmaat, A.J., and Verhaagen, J. (1998). Evidence for a role of the chemorepellent semaphorin III and its receptor neuropilin-1 in the regeneration of primary olfactory axons. J. Neurosci *18*, 9962-9976.

S4. Andl, T., Reddy, S.T., Gaddapara, T., and Millar, S.E. (2002). WNT signals are required for the initiation of hair follicle development. Dev Cell *2*, 643-653.

S5. Licht, T., Goshen, I., Avital, A., Kreisel, T., Zubedat, S., Eavri, R., Segal, M., Yirmiya, R., and Keshet, E. (2011). Reversible modulations of neuronal plasticity by VEGF. Proc. Natl. Acad. Sci. USA *108*, 5081-5086.

S6. Ciani, L., Marzo, A., Boyle, K., Stamatakou, E., Lopes, D.M., Anane, D., McLeod, F., Rosso, S.B., Gibb, A., and Salinas, P.C. (2015). Wnt signalling tunes neurotransmitter release by directly targeting Synaptotagmin-1. Nat Commun *6*, 8302.

S7. Bourne, J.N., and Harris, K.M. (2008). Balancing structure and function at hippocampal dendritic spines. Annu Rev Neurosci *31*, 47-67.

S8. Schikorski, T., and Stevens, C.F. (1997). Quantitative ultrastructural analysis of hippocampal excitatory synapses. J. Neurosci *17*, 5858-5867.

S9. Malleret, G., Haditsch, U., Genoux, D., Jones, M.W., Bliss, T.V., Vanhoose, A.M., Weitlauf, C., Kandel, E.R., Winder, D.G., and Mansuy, I.M. (2001). Inducible and reversible enhancement of learning, memory, and long-term potentiation by genetic inhibition of calcineurin. Cell *104*, 675-686.

S10. Irvine, E.E., Drinkwater, L., Radwanska, K., Al-Qassab, H., Smith, M.A., O'Brien, M., Kielar, C., Choudhury, A.I., Krauss, S., Cooper, J.D., et al. (2011). Insulin receptor substrate 2 is a negative regulator of memory formation. Learn Mem *18*, 375-383.
