# Supplementary material for: Reversal of Synapse Degeneration by Restoring Wnt Signaling in the Adult Hippocampus
Source: Curr Biol. 2016 Oct 10;26(19):2551–61. doi: 10.1016/j.cub.2016.07.024 (PMC5070786; doi:10.1016/j.cub.2016.07.024)
Supplement: Document S2. Article plus Supplemental Information [file mmc2.pdf]

Article

Current Biology

Reversal of Synapse Degeneration by Restoring Wnt Signaling in the Adult Hippocampus

Graphical Abstract

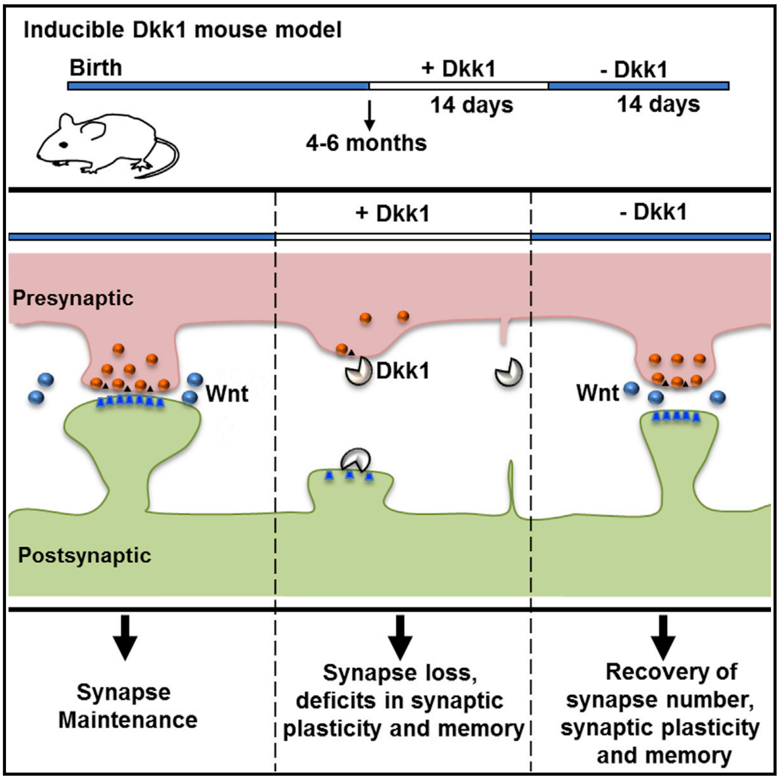

Authors

Aude Marzo, Soledad Galli, Douglas Lopes, ..., Francesca Cacucci, Alasdair Gibb, Patricia C. Salinas

Correspondence

p.salinas@ucl.ac.uk

In Brief

Deficiency in Wnt signaling has been implicated in Alzheimer's disease. Marzo et al. elucidate the impact of the Wnt antagonist Dkk1 in the adult hippocampus, showing synapse loss and defects in synaptic plasticity and long-term memory. They also reveal that cessation of Dkk1 expression induces synapse regeneration and recovery of long-term memory.

Highlights

- Wnt signaling is required for synapse integrity in the adult hippocampus
- Dkk1 induces synapse loss and deficits in synaptic plasticity and long-term memory
- Dkk1 disassembles synapses by activating the Gsk3 and Rock pathways
- Synapse loss and memory defects are reversible by reactivation of the Wnt pathway

Marzo et al., 2016, Current Biology *26*, 2551–2561
October 10, 2016 © 2016 The Author(s). Published by Elsevier Ltd.
http://dx.doi.org/10.1016/j.cub.2016.07.024

Current Biology
Article

Reversal of Synapse Degeneration by Restoring Wnt Signaling in the Adult Hippocampus

Aude Marzo,[1,3] Soledad Galli,[1,3] Douglas Lopes,[1,3] Faye McLeod,[1,3] Marina Podpolny,[1] Margarita Segovia-Roldan,[1] Lorenza Ciani,[1] Silvia Purro,[1] Francesca Cacucci,[2] Alasdair Gibb,[2] and Patricia C. Salinas[1,4,*]
[1]Department of Cell and Developmental Biology
[2]Department of Neuroscience, Physiology, and Pharmacology
University College London, London WC1E 6BT, UK
[3]Co-first author
[4]Lead Contact
*Correspondence: p.salinas@ucl.ac.uk
http://dx.doi.org/10.1016/j.cub.2016.07.024

SUMMARY

Synapse degeneration occurs early in neurodegenerative diseases and correlates strongly with cognitive decline in Alzheimer's disease (AD). The molecular mechanisms that trigger synapse vulnerability and those that promote synapse regeneration after substantial synaptic failure remain poorly understood. Increasing evidence suggests a link between a deficiency in Wnt signaling and AD. The secreted Wnt antagonist Dickkopf-1 (Dkk1), which is elevated in AD, contributes to amyloid-β-mediated synaptic failure. However, the impact of Dkk1 at the circuit level and the mechanism by which synapses disassemble have not yet been explored. Using a transgenic mouse model that inducibly expresses Dkk1 in the hippocampus, we demonstrate that Dkk1 triggers synapse loss, impairs long-term potentiation, enhances long-term depression, and induces learning and memory deficits. We decipher the mechanism involved in synapse loss induced by Dkk1 as it can be prevented by combined inhibition of the Gsk3 and RhoA-Rock pathways. Notably, after loss of synaptic connectivity, reactivation of the Wnt pathway by cessation of Dkk1 expression completely restores synapse number, synaptic plasticity, and long-term memory. These findings demonstrate the remarkable capacity of adult neurons to regenerate functional circuits and highlight Wnt signaling as a targetable pathway for neuronal circuit recovery after synapse degeneration.

INTRODUCTION

Synapse loss and dysfunction are an early occurrence in several neurodegenerative conditions, including Alzheimer's disease (AD). Synapse vulnerability strongly correlates with cognitive decline before detectable neuronal death [1, 2] and might contribute to the subsequent neuronal degeneration. Surprisingly, little is known about the molecular mechanisms that trigger synapse vulnerability in neurodegenerative diseases and even less about how this process can be prevented or reversed.

Increasing evidence suggests that deficient canonical Wnt signaling contributes to AD pathogenesis. Wnts are secreted proteins that modulate several aspects of brain development and function, including synapse formation, synaptic transmission, experience-mediated synaptic remodeling, and adult neurogenesis [3–7]. Genome-wide association studies (GWASs) have revealed a link between genetic variants of the Wnt co-receptor LRP6, which are associated with decreased canonical Wnt signaling activity, and late onset AD [8, 9]. Loss of function of LRP6 in hippocampal neurons results in synaptic defects, cell death, and exacerbation of amyloid deposition in a mouse model of AD [10]. Importantly, the secreted protein Dickkopf-1 (Dkk1), which blocks canonical Wnt-Gsk3 signaling by sequestering the LRP6 receptor [11, 12], is elevated in post-mortem brains from AD patients and in AD animal models [13–15]. In addition, oligomers of amyloid-β (Aβ), the main component of amyloid plaques in AD, induce Dkk1 expression in cultured neurons and in brain slices [13, 16, 17]. Dkk1 disassembles excitatory synapses in a similar manner to Aβ in cultured hippocampal neurons [17]. Importantly, blockade of Dkk1 with neutralizing antibodies protects synapses from Aβ-mediated disassembly [17]. Collectively, these results suggest that Dkk1-mediated deficiency of Wnt signaling could contribute to synapse vulnerability. However, the impact of Dkk1 on hippocampal circuits, which are severely affected in AD, and its mechanism of action have not been explored.

Restoration of synaptic function after substantial synapse loss is crucial for the treatment of neurodegenerative diseases, as diagnosis is often obtained after significant damage has occurred. Although some downstream targets of Aβ have been identified [18–21], only a limited number of studies has shown the ability of these molecules to fully restore function after significant synapse degeneration [18, 20]. Thus, the identity of the signaling pathways that could restore synapse function remains poorly understood.

Here, we demonstrate a critical role for Wnt signaling in synapse stability and synaptic plasticity in the adult hippocampus. Using a transgenic mouse model that allows inducible expression of Dkk1, we investigated the contribution of deficient Wnt signaling to synapse function in the adult hippocampus without compromising embryonic and postnatal development. Inducible Dkk1 expression triggers disassembly of excitatory

This is an open access article under the CC BY license (http://creativecommons.org/licenses/by/4.0/).

synapses, defects in long-term potentiation (LTP), and facilitation of long-term depression (LTD). Consistent with these synaptic plasticity changes, hippocampal-mediated long-term memory is impaired. These synaptic deficits occur in the absence of cell death or changes in the stem cell niche. Thus, the Dkk1 inducible (iDkk1) mouse is a good model system to study synapse degeneration in the absence of cell loss. Our studies reveal that Dkk1 induces synapse degeneration through the combined activation of Gsk3 and a novel target of Dkk1, the RhoA-Rock pathway. Notably, we found that reactivation of Wnt signaling, by cessation of Dkk1 expression, results in full recovery of synapse structure, synaptic plasticity, and long-term memory. In summary, our studies demonstrate that deficient Wnt signaling leads to synapse loss in vivo as observed at early stages of Aβ-mediated pathogenesis and reveal the remarkable regenerative capacity of neurons in the adult hippocampus to assemble synapses within functional circuits. Our work highlights the importance of Wnt signaling in this process and identifies new targetable molecules for protecting synapses from degeneration.

RESULTS

Inducible Dkk1-Expressing Mice as a Model for Wnt Deficiency in the Adult Brain

To investigate the contribution of Wnt signaling to synapse maintenance in the adult hippocampus, we took advantage of a transgenic mouse model where expression of a potent and specific secreted Wnt antagonist, Dkk1, is controlled under the tetracycline-inducible system and CaMKII promoter [22]. Expression of Dkk1 is induced in adult mice by administration of doxycycline, bypassing any potential deleterious effects of deficient Wnt signaling during embryogenesis and postnatal development, stages when Wnt signaling plays a critical role [12, 23–25]. Mice carrying the Dkk1 coding region under the control of the doxycycline responsive element (tetO) [26] were crossed to mice carrying the tetracycline-controlled transactivator (rtTA2S; rtTA hereafter) downstream of the CaMKIIα promoter (CaMKII hereafter) [27]. Dkk1 expression was induced in adult (3–6 months of age) double transgenic mice (iDkk1) by administration of doxycycline into their diet for 2 weeks for full induction of the CaMKII-rtTA/tetO system [28] (Figure 1A).

Dkk1 expression was detected by RT-PCR in the hippocampus of adult iDkk1 mice fed with doxycycline, but not in control littermates fed with doxycycline or in iDkk1 mice not fed with doxycycline (Figure 1B). Dkk1 mRNA expression could be detected after 3 days of induction and sustained for the duration of doxycycline administration (Figure 1B). Thus, expression of Dkk1 is tightly regulated by doxycycline in iDkk1 mice. Most of our studies were performed after 2 weeks induction (unless otherwise indicated) when expression of Dkk1 was clearly observed by in situ hybridization (Figure 1C) in a large number of principal hippocampal neurons in the CA1, CA3, and dentate gyrus (DG). These mice developed normally and had similar weight to control mice (Figure S1).

Dkk1 Does Not Affect Cell Death or the Stem Cell Niche in the Adult Hippocampus

Deficiency in Wnt signaling has been implicated in regulating cell viability and the stem cell niche in the adult hippocampus [13, 29, 30]. We therefore examined these two aspects in the hippocampus of adult iDkk1 mice expressing Dkk1 for 2 weeks. TUNEL assays and the levels of cleaved caspase 3 revealed no changes in cell death (Figures S2A–S2C). The number of NeuN-positive neurons was not altered (Figure 1D) after 14 days or after 3.5 months of Dkk1 induction. These findings demonstrate that induced Dkk1 expression in the adult hippocampus does not affect cell viability.

Next, we examined possible changes in the stem cell niche in the adult DG, the main source of neuronal stem cells in the hippocampus. The number of newly born neurons, labeled by the specific marker doublecortin (Dcx) [31], did not change upon Dkk1 induction (Figure S2D). Consistent with no changes in cell number, the overall morphology of the brain and the architecture of the hippocampus were normal (Figures S2A and S2B). Thus, induced expression of Dkk1 in the adult hippocampus does not affect cell viability or the stem cell niche.

Wnt Signaling Blockade in the Adult Hippocampus Results in Long-Term Memory Deficits

The hippocampus plays a role in emotional and cognitive functions, such as anxiety, learning, and memory [32, 33]. We investigated the impact of Dkk1 expression in these processes. The exploratory activity and anxiety level, evaluated through an open-field and elevated plus maze, were identical in iDkk1 mice and controls (Figures S3A and S3B). In addition, no defects were observed in the swimming speed and traveled distance in a Morris water maze (MWM) (Figure S3C), demonstrating that Dkk1 expression does not affect motor function and hippocampal-dependent emotional behaviors.

Next, we investigated short-term memory using the discrete trial version of the spontaneous alternation T-maze test (30-s delay) [34]. This task depends on the animals' natural tendency to alternate and enter the previously unvisited arm. Both control and iDkk1 mice alternated between the two arms above chance (Figure S3D), indicating that short-term memory is unaffected in iDkk1 mice. We then evaluated hippocampus-dependent spatial reference learning and long-term memory using the MWM test [35–37]. Mice were first trained on the cued version of the task (platform marked by a visible flag). No difference in the time required to reach the visible platform was observed between control and iDkk1 mice (Figure 1E), demonstrating that iDkk1 mice have no visual and procedural skills defects. Subsequently, mice were trained over 5 days to locate an invisible platform. The platform was removed during two probe tests (before the 4th day and 24 hr after the 5th day of training). iDkk1 mice took twice as long as controls to find the hidden platform on the 3rd and 4th days of training (Figure 1E), demonstrating an inability to remember the location of the platform. Similarly, during the first probe test (probe I), iDkk1 mice spent less time in the target quadrant and crossed the virtual platform location significantly fewer times than controls (Figures 1F and 1G), demonstrating impaired reference memory acquisition. Interestingly, after two further training days, iDkk1 mice reached the same performance level as control mice (probe II; Figures 1F and 1G), suggesting that additional training can overcome this memory deficit, as shown in some AD mouse models [38–40]. Thus, deficient Wnt signaling in the adult hippocampus leads to deficits in spatial memory acquisition.

To extend our study of memory-related hippocampal function, we used a single-trial contextual fear-conditioning paradigm [41,

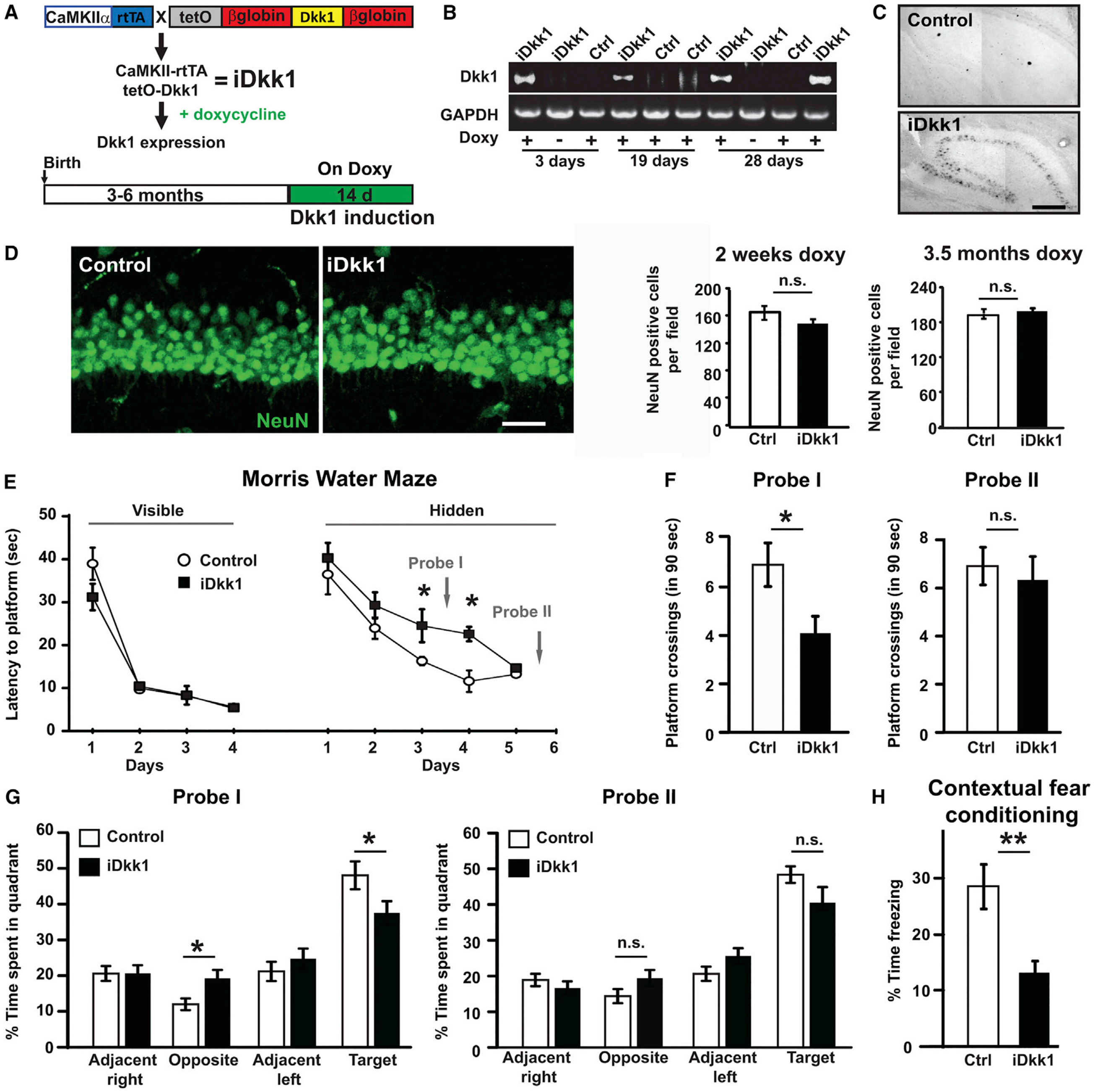

Figure 1. Expression of Dkk1 in Adult Hippocampus Induces Defects in Long-Term Memory

(A) Top: schematic of doxycycline-induced Dkk1 expression. Mice carrying the rtTA gene under the CaMKIIα promoter are crossed with tetO-Dkk1 mice to generate double transgenic animals (iDkk1). Bottom: schematic representation of doxycycline feeding schedule in adult mice (see Figure S1).

(B) RT-PCR for Dkk1 in hippocampus of adult iDkk1 and control mice with or without doxycycline administration (Doxy).

(C) In situ hybridization for Dkk1 mRNAs in adult hippocampus of iDkk1 and control mice. The scale bar represents 250 μm.

(D) Images and quantification of NeuN-positive CA1 neurons in control and iDkk1 mice fed with doxycycline for 14 days. Quantification of NeuN-positive CA1 neurons is also shown after 3.5 months of diet containing doxycycline (ANOVA; four mice per genotype per condition). The scale bar represents 50 μm (see also Figure S2).

(E) Escape latency in the Morris water maze (MWM) (*$p < 0.05$; repeated-measures ANOVA; 11 control and 12 iDkk1 mice; see also Figure S3).

(F) Number of platform crossings in the MWM during probe I (left) or during probe II (right; *$p < 0.05$; Student's t test).

(G) Time spent in each quadrant during probe I (left) and during probe II (right; *$p < 0.05$; Student's t test).

(H) Percentage of freezing time evaluated 24 hr after the foot shock (**$p \leq 0.01$; ANOVA; eight control and seven iDkk1 mice).

Data are represented as mean ± SEM.

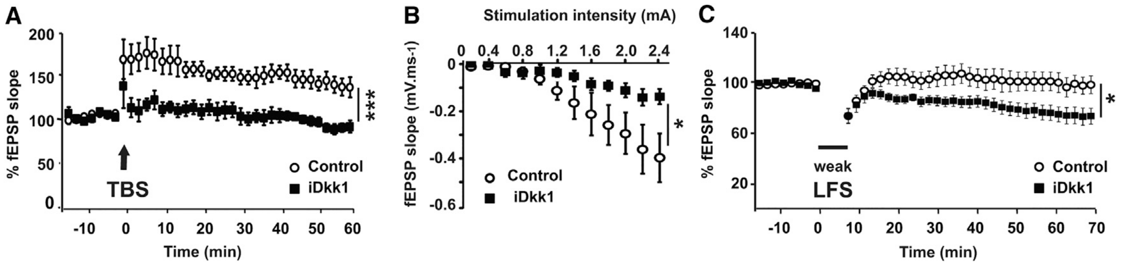

Figure 2. Dkk1 Impairs LTP and Basal Synaptic Transmission and Enhances LTD in the Adult Hippocampus

(A) LTP was induced by theta-burst protocol (TBS) on Schaffer collateral axons (ten slices from six controls and eight slices from seven iDkk1 mice; ***$p \leq 0.001$; repeated-measures ANOVA).

(B) Input-output curve shows fEPSP slope in CA1 in response to different stimulus intensity of Schaffer collateral axons (12 slices from eight control and 11 slices from seven iDkk1 mice; *$p < 0.05$; repeated-measures ANOVA).

(C) A weak low-frequency stimulation (weak LFS) induces short-term depression in control slices and LTD in iDkk1 slices (11 slices from six controls and nine slices from five iDkk1 mice; *$p < 0.05$; repeated-measures ANOVA; see also Figure S4).

Data are represented as mean ± SEM.

42]. We compared the percentage of freezing time displayed by mice when re-introduced into the conditioning chamber after having associated the context to a foot shock. iDkk1 mice showed a considerably reduced freezing time compared to controls upon reintroduction to the conditioning chamber 24 hr after the context/shock single pairing (Figure 1H). This result indicates that iDkk1 mice were unable to form a strong association between the contextual cues and the foot shock. Together, our behavioral studies show that iDkk1 mice exhibit deficits in hippocampal-dependent long-term memory.

Deficient Wnt Signaling Impairs Basal Synaptic Transmission and Synaptic Plasticity

Changes in long-term memory have been correlated with changes in long-term synaptic plasticity (i.e., LTP and LTD) [43–45]. We therefore investigated the ability of iDkk1 mice to express LTP at Schaffer collateral (SC)-CA1 synapses. A theta-burst stimulation (TBS) protocol was chosen as it mimics hippocampal activity during spatial learning [46]. TBS induced a 40% potentiation in control mice, whereas in iDkk1 mice it failed to potentiate these synapses (Figure 2A), demonstrating that Wnt blockade in the adult hippocampus results in the absence of TBS-induced LTP.

This defect could be due to a decreased connectivity, as a minimal number of synapses is required to promote LTP induction as defined as cooperativity [47]. Analyses of input-output curves at the SC-CA1 synapses revealed a defect at the strongest intensities of stimulation in iDkk1 mice, as the field excitatory postsynaptic potential (fEPSP) slope reached only half the magnitude of control animals (Figure 2B). Thus, CA1 synaptic connectivity is affected by Dkk1 expression.

LTD is crucial to synaptic function, and its modulation by Wnt signaling remains unknown. To examine the impact of Dkk1 on LTD, we used a protocol that effectively induces LTD in adult mice with a strong low-frequency stimulation (LFS) consisting of two trains of 900 pulses at 2 Hz. With this protocol, we observed a 20%–30% depression at the SC-CA1 synapses in both control and iDkk1 animals (Figure S4). We therefore decided to use a sub-threshold LFS (weak LFS) protocol, which has been shown to unmask enhanced LTD after exposure to Aβ [48, 49]. We used a weak LFS protocol, consisting of a single train of 900 pulses at 2 Hz, which induced a short-term but no long-term depression in control animals (Figure 2C) [50]. In contrast to control animals, this weak LFS induced LTD in iDkk1 mice (Figure 2C). This is the first demonstration that Wnt signaling contributes to LTD expression. Thus, Wnt deficiency induced by Dkk1 expression facilitates LTD and blocks LTP at SC-CA1 synapses in the adult hippocampus.

Dkk1 Triggers Degeneration of Excitatory Synapses in the Adult Hippocampus

To determine the impact of Dkk1 expression on synapse stability, we measured excitatory synapses by the co-localization of pre- and postsynaptic markers (vGlut1 and PSD95, respectively) in the CA1 stratum radiatum. iDkk1 mice exhibited fewer excitatory synapses (~40% decrease; Figure 3A). Consistently, we observed a similar decrease in the number of asymmetric (i.e., excitatory) synapses in the CA1 stratum radiatum by electron microscopy (Figure 3B). Thus, Dkk1 triggers the degeneration of glutamatergic synapses in the adult hippocampus. To evaluate neuronal connectivity, we recorded miniature excitatory postsynaptic currents (mEPSCs) using whole-cell patch-clamp recordings from CA1 neurons. Although no changes in mEPSC amplitude were observed, we found a significant decrease in mEPSC frequency (~40%) in iDkk1 mice (Figures 3C and 3D), consistent with a decrease in excitatory synapse number.

In contrast, induced Dkk1 expression did not affect the number of inhibitory synapses in the CA1 region, as determined by co-localization of the pre- and postsynaptic markers vGat and gephyrin (Figure 4A). Consistently, the amplitude and frequency of miniature inhibitory postsynaptic currents (mIPSCs) in hippocampal CA1 neurons were unaffected by Dkk1 expression (Figure 4B). Thus, Dkk1 specifically affects the integrity of excitatory synapses without altering inhibitory synapses.

Dkk1 Triggers Synaptic Disassembly by Blocking Canonical Wnt Signaling and Activating the RhoA-Rock Pathway

Dkk1 is a known specific Wnt antagonist that blocks canonical Wnt signaling [11, 12]. Wnt ligands bind to Frizzled (Fz) receptors

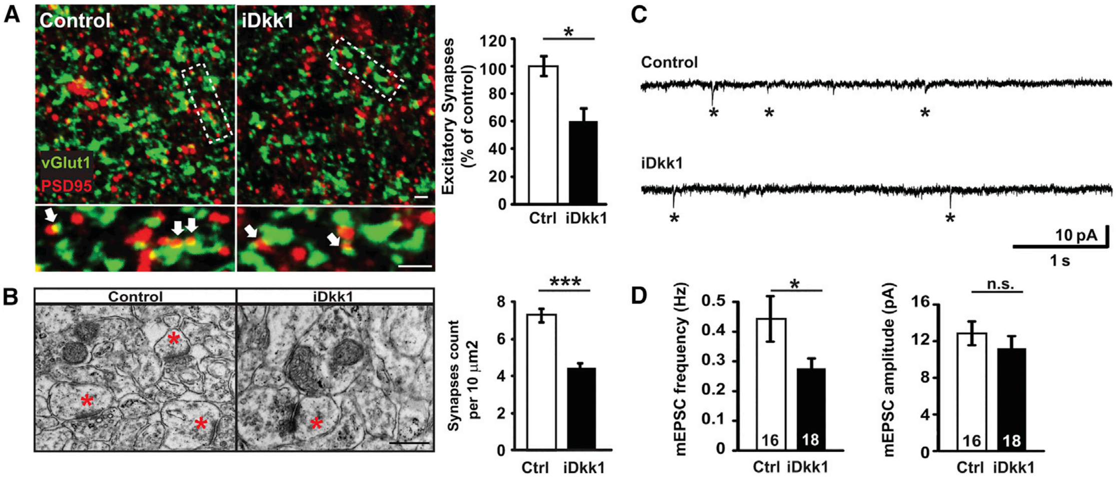

Figure 3. Blockade of Wnt Signaling Triggers Excitatory Synapse Loss and Dysfunction in the Adult Hippocampus in iDkk1 Mice

(A) Confocal images from hippocampal CA1 show excitatory synapses (co-localized pre- and postsynaptic markers; vGlut1 and PSD95 puncta, respectively; white arrows). The scale bars represent 2 µm. Quantification is shown on the right-hand side (*$p < 0.05$; Kruskal-Wallis test; six mice per genotype).

(B) Electron micrographs show asymmetric synapses (red stars) in the CA1 stratum radiatum. The scale bar represents 0.5 µm. Quantification is shown on the right-hand side (***$p \leq 0.001$; ANOVA; five mice per genotype).

(C) Representative mEPSC traces recorded at −60 mV from CA1 cells.

(D) Quantification of mEPSC frequency and amplitude. Numbers inside bars indicate the number of cells recorded from at least seven mice per genotype (*$p < 0.05$; Mann-Whitney test for frequency; Student's t test for amplitude).

Data are represented as mean ± SEM.

and the co-receptors LRP6, resulting in the inhibition of Gsk3β-mediated phosphorylation and stabilization of β-catenin, which translocates to the nucleus and activates transcription [51] (Figure S5). In contrast, in the presence of Dkk1, binding of Wnts to Fz/LRP6 is blocked, resulting in enhanced Gsk3β-mediated β-catenin degradation by the proteasome pathway (Figure S5) [12, 51]. Thus, Dkk1 effectively blocks the function of several Wnts that signal through the LRP6 receptor. To investigate the impact of Dkk1 expression in canonical Wnt signaling, we evaluated β-catenin levels. Indeed, expression of Dkk1 resulted in fewer β-catenin puncta in the CA1 stratum radiatum of iDkk1 mice (Figures 5A and 5B), indicating that Dkk1 blocks the canonical Wnt-β-catenin pathway. Co-localization with the synaptic marker vGlut1 showed that most β-catenin puncta were extrasynaptic, indicating that the loss of β-catenin induced by Dkk1 was not due to synapse loss. These results suggest that Dkk1 expression blocks canonical Wnt signaling in the adult hippocampus.

Next, we evaluated whether Dkk1-mediated synaptic loss is due to blockade of canonical Wnt signaling. We used the specific Gsk3 inhibitor BIO (6-bromoindirubin-3′-oxime), which activates the Wnt pathway downstream of Dkk1 [52, 53]. Using a concentration of BIO, which does not affect synapse number on its own (Figures 5C and 5D), we found that this Gsk3 inhibitor partially occluded Dkk1-induced synapse disassembly (Figures 5C and 5D), suggesting that Dkk1 induces synapse loss through blockade of the Wnt-Gsk3β pathway but additional pathways might be involved. Dkk1 is mostly known as a specific and potent inhibitor of the Wnt-Gsk3β pathway; however, some studies have suggested that Dkk1 could activate non-canonical Wnt pathways [16, 54–56]. Although a role for the RhoA-Rock pathway in Dkk1 has not been reported in neurons, this cascade is of particular interest because it has been implicated in synaptic plasticity, learning, and memory and in Aβ-mediated synapse loss [57–59]. We therefore examined the role of this pathway in Dkk1-mediated synapse degeneration. Exposure to Y27632, a specific Rock inhibitor, partially prevented Dkk1-mediated synapse loss (Figures 5C and 5E). Given the partial protection by both Gsk3β inhibition and Rock inhibition on Dkk1-mediated synapse degeneration, we examined the combined effect of Gsk3β and Rock inhibitors and found complete blockade of Dkk1-induced synapse loss (Figures 5C and 5F). These results demonstrate a novel role for RhoA-Rock pathway in Dkk1 function and suggest that Dkk1 promotes synapse disassembly by blocking canonical Wnt signaling and activating the RhoA-Rock pathway.

Synaptic Loss, Plasticity Defects, and Behavioral Impairment Are Reversible

Diagnosis of neurodegenerative diseases is often made after substantial loss of synaptic connectivity has occurred. Thus, understanding the reversible nature of synaptic degeneration is crucial for developing therapies for the treatment of cognitive impairments in neurodegenerative diseases. We therefore examined whether Dkk1-mediated synapse loss and network dysfunction is reversible. We performed in vivo on-off experiments (Figure 6A), in which Dkk1 expression was induced for 2 weeks with doxycycline (On Doxy), followed by withdrawal of doxycycline for a further 2 weeks (Off Doxy). RT-PCR revealed that Dkk1 was expressed during the "on" period, but not after the "off" period, confirming that Dkk1 expression is tightly regulated by doxycycline (Figure 6B). Remarkably, the number of excitatory synapses fully recovered to control levels after doxycycline withdrawal (Figures 6C and 6D). These results

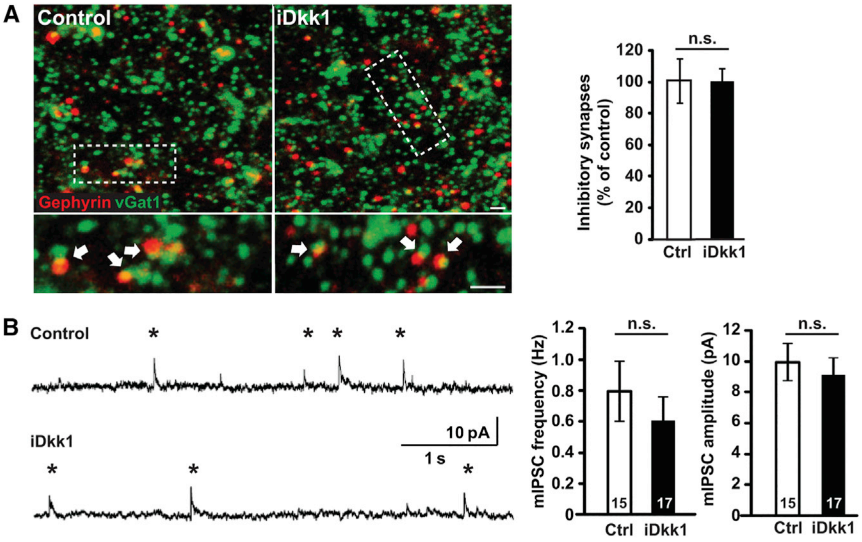

Figure 4. Dkk1 Does Not Affect Inhibitory Synapses in the Hippocampus in iDkk1 Mice

(A) Confocal images of adult CA1 stratum radiatum show the presence of inhibitory synapses identified by co-localized presynaptic vGat and postsynaptic Gephyrin puncta (white arrows). The scale bars represent 2 μm. Quantification is shown on the side (Kruskal-Wallis test; three mice per genotype).

(B) Representative mIPSC traces recorded at 0 mV from CA1 cells in acute hippocampal slices and quantification of mIPSC frequency and amplitude. Numbers inside bars indicate the number of cells recorded from at least seven mice per genotype (Mann-Whitney test for frequency; Student's t test for amplitude).

Data are represented as mean ± SEM.

demonstrate that, even after significant degeneration, the number of synaptic connections can be restored when Dkk1 expression is turned off in the adult hippocampus.

We then evaluated whether defects in basal transmission, long-term plasticity, and long-term memory could be reversed in iDkk1 mice. We found that cessation of Dkk1 expression resulted in full recovery of basal synaptic transmission as indicated by the overlapping input-output curves from control and iDkk1 mice (Figure 6E). Notably, TBS fully induced LTP in iDkk1 mice after termination of Dkk1 expression (Figure 6F). Moreover, weak LFS induced short-term depression without inducing LTD in both control and iDkk1 mice (Figure 6G). Finally, using the contextual fear-conditioning test, we found that turning off Dkk1 expression in iDkk1 mice completely recovers their ability to form long-term memory, as the percentage of freezing time was similar to control mice (Figure 6H). Taken together, these studies show the remarkable capacity of the adult hippocampus to regenerate synapses that integrate into functional neuronal circuits. They also demonstrate that synapse degeneration can be reversed in the adult mouse brain by modulating Wnt signaling.

DISCUSSION

Here, we report that deficiency in Wnt signaling by inducibly expressing the specific Wnt antagonist Dkk1 in the adult hippocampus triggers the loss of excitatory synapses in CA1 neurons, impairs synaptic plasticity, and alters hippocampal-dependent function. These defects occur in the absence of cell death and require the combined activation of Gsk3β and Rock. Notably, Dkk1-induced synaptic defects are fully reversed upon cessation of Dkk1 expression. Our findings demonstrate that iDkk1 mice provide a unique model system to study the in vivo impact of deficient Wnt signaling on synapse vulnerability and to elucidate the molecular mechanisms that contribute to synapse regeneration after substantial synapse loss and dysfunction.

In the adult hippocampus, Dkk1 expression blocks Wnt signaling without affecting cell viability or the stem cell niche. Previous studies have shown that Dkk1 can promote cell death in models of AD, epilepsy, and ischemia [13, 29, 60, 61] and affect adult neurogenesis by modulating the generation of immature neurons in the adult DG [30]. However, we found no evidence of increased cell death or an effect on the number of newborn neurons in the adult hippocampus of iDkk1 mice. This could be attributed to low levels of Dkk1 expression after 2 weeks induction of this protein. Given the direct effect of Wnts on synapses [62–64], our results suggest that Dkk1 induces synaptic vulnerability by directly targeting synapses.

Blockade of Wnt signaling with Dkk1 specifically affects excitatory synapses in the adult hippocampus, resulting in decreased mEPSC frequency and reduced excitatory synaptic transmission. In contrast, Dkk1 does not affect the number of inhibitory synapses or mIPSC frequency and amplitude. In the adult striatum, Dkk1 also induces the loss of excitatory synapses [22]. Together, these results highlight the crucial role for Wnt signaling in the maintenance of functional excitatory synapses in the adult brain. Although the mechanism by which Dkk1 specifically affects excitatory, but not inhibitory, synapses remains unknown, recent studies showed that LRP6 is predominantly present at excitatory synapses [65] and that deficiency in LRP6 affects excitatory synapses in the hippocampus [10]. These results suggest that Dkk1 acts through LRP6, which is upstream of the Wnt-Gsk3β pathway. Consistent with the inhibition of this pathway, the number of β-catenin puncta decreases in hippocampal CA1 following Dkk1 expression. Thus, induced expression of Dkk1 compromises the canonical Wnt pathway.

Dkk1 induces synapse degeneration by modulating the Wnt-Gsk3β and the Rock pathways. Our studies reveal that inhibiting Gsk3β with BIO partially blocks Dkk1-mediated synapse disassembly, suggesting that additional pathways might be involved. Previous studies showed that activation of the RhoA-Rock pathway leads to spine loss and mediates Aβ-induced synapse loss [57–59]. Interestingly, we found that Rock inhibition partially blocks Dkk1-induced synapse degeneration. In contrast, inhibition of both Gsk3β and Rock completely protects synapses against Dkk1. Therefore, we have identified Rock as a novel downstream target for Dkk1. How Dkk1 activates Gsk3β and Rock pathways is unknown. Both signaling cascades could

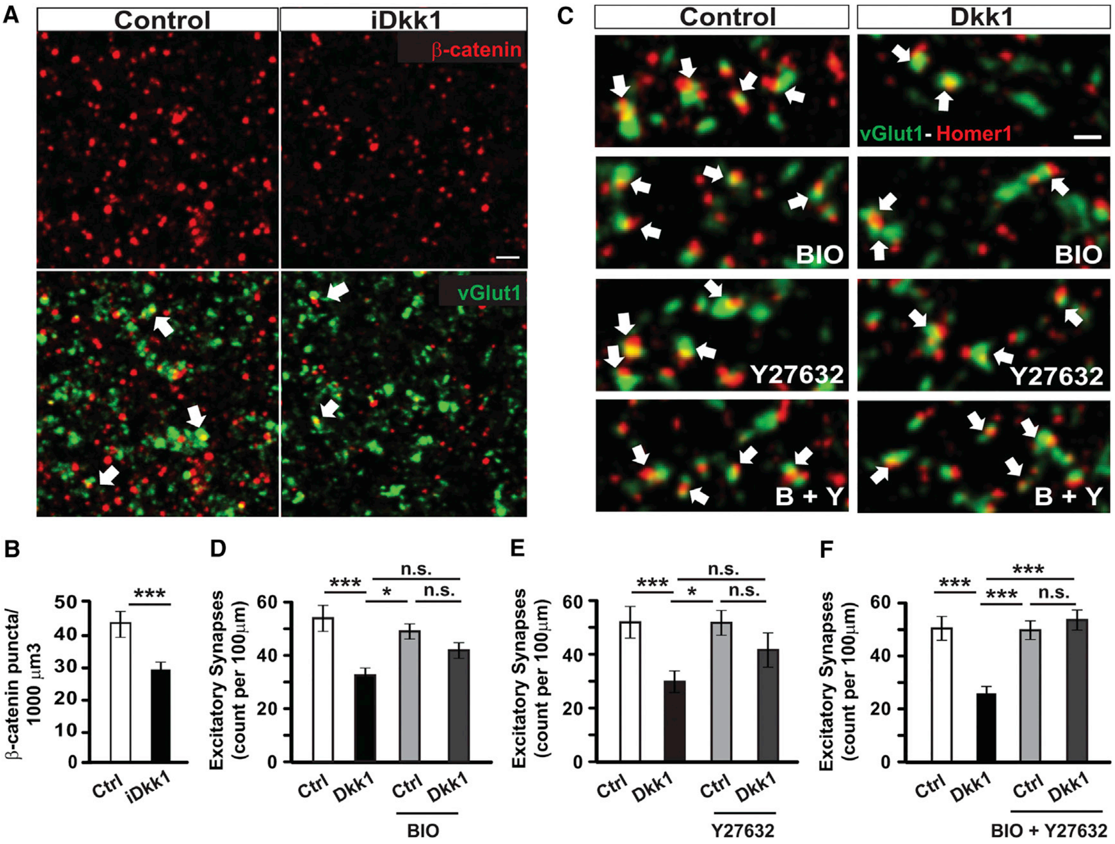

Figure 5. Dkk1 Induces Synapse Loss through Blockade of Canonical Wnt-Gsk3 Pathway and Activation of the RhoA-Rock Pathway

(A) Distribution of β-catenin and vGlut1 puncta in the hippocampal CA1 stratum radiatum as indicated on the right corner. White arrows indicate that only few β-catenin puncta co-localize with vGlut1. The scale bar represents 2 μm (see also Figure S5).

(B) Graph shows quantification of β-catenin puncta (***$p \leq 0.001$; ANOVA; four mice per genotype).

(C) Confocal images show the presence of excitatory synapses (co-localized vGlut1 and Homer1 puncta; white arrows) in mature hippocampal neurons exposed to control or Dkk1 and specific Gsk3 and Rock inhibitors as indicated. The scale bar represents 2 μm.

(D–F) Quantification of excitatory synapses per 100 μm dendrite after treatment with Dkk1 and with BIO, a Gsk3 inhibitor (D), with a Rock inhibitor, Y27632 (E), or with both BIO and Y27632 (F; *$p < 0.05$; one-way ANOVA test; n = 3 independent experiments per condition).

Data are represented as mean ± SEM.

influence the stability of the synapse by modulating different targets, such as β-catenin and microtubules in the case of Gsk3β or the actin cytoskeleton through the Rock pathway. Alternatively, both pathways could interact as recently reported for the role of Wnts in cell migration [66]. Future studies will elucidate the downstream events by which these two pathways contribute to Dkk1-mediated synapse vulnerability.

Induced Dkk1 expression affects long-term plasticity and memory. iDkk1 mice exhibit impaired hippocampus-dependent function as demonstrated by defects in contextual fear memory and spatial learning and memory. These results are in agreement with a previous study suggesting a role for Wnt signaling in memory [16, 67]. Memory deficits have been associated with defects in long-term plasticity in the hippocampus [68–70]. Consistently, iDkk1 mice exhibit a significant impairment in LTP, a defect that could be due to the loss of 40% of excitatory synapses [47] and/or to the impaired ability of remaining synapses to respond to LTP induction. We also demonstrate a novel function for Wnt signaling in LTD. Previous studies showed that Gsk3β activation suppresses LTP [71] and enhances LTD [72], suggesting a role for Gsk3β downstream of Dkk1-mediated synaptic dysfunction.

Understanding the molecular pathways that promote the regeneration of synapses that integrate into networks is crucial for developing effective therapies to promote functional recovery. Here, we report that synapse loss, defects in synaptic plasticity, and memory deficits can be fully restored in iDkk1 mice after cessation of Dkk1 expression. Our findings demonstrate the remarkable capacity of adult neurons to regenerate functional circuits after substantial synapse loss and highlights that Wnt signaling is a targetable pathway in neurodegenerative diseases.

EXPERIMENTAL PROCEDURES

Animals

Experiments were performed according to the Animals Scientific procedures Act UK (1986). Double transgenic mice (iDkk1) were obtained as described in [22]. Adult (3–6 months old) iDkk1 and control mice (tetO-Dkk1, CaMKIIα-rtTA2, or wild-type littermates) were fed with pellets containing 6 mg/kg doxycycline (Datesand Group) ad libitum for 2 weeks, unless otherwise indicated. For the

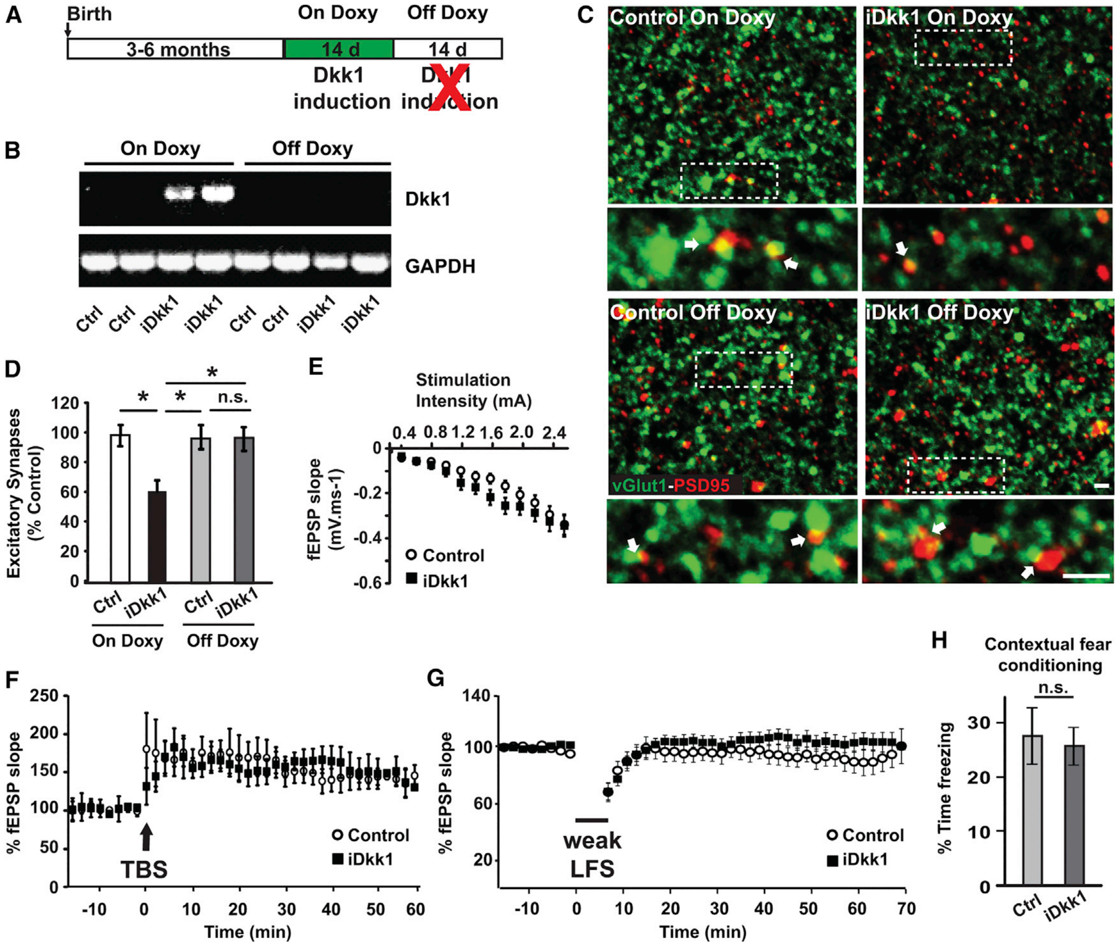

Figure 6. Synapse Loss, Long-Term Plasticity, and Memory Defects Are Reversible

(A) Schematic representation of doxycycline feeding schedule in adult mice.

(B) RT-PCR for Dkk1 in mice fed with doxycycline for 2 weeks (On Doxy) or after subsequent 2 weeks without doxycycline (Off Doxy).

(C) Images of hippocampal CA1 show excitatory synapses (co-localized vGlut1 and PSD95 puncta; white arrows). The scale bar represents 2 μm.

(D) Quantification of excitatory synapses (*p < 0.05; Kruskal-Wallis test; six mice per genotype).

(E) Input-output curves show no difference between control and iDkk1 mice after doxycycline withdrawal (nine slices from five controls and 11 slices from seven iDkk1 mice; repeated-measures ANOVA).

(F) TBS-induced LTP in control and iDkk1 mice after doxycycline withdrawal (seven slices from six controls and nine slices from six iDkk1 mice; repeated-measures ANOVA).

(G) Weak LFS failed to induce LTD at the SC-CA1 synapses of control or iDkk1 mice after doxycycline withdrawal (nine slices from seven controls and eight slices from five iDkk1 mice; repeated-measures ANOVA).

(H) Percentage of freezing time evaluated 24 hr after the foot shock (15 control and 14 iDkk1 mice).

Data are represented as mean ± SEM.

on-off experiment, 2 weeks of doxycycline feeding was followed by 2 weeks of feeding with the original diet. Males were used for electrophysiological and behavioral experiments, whereas both genders were used for cellular biology experiments. See the Supplemental Experimental Procedures for more details.

Hippocampal Culture, Cell Transfection, and Drug Treatment

Hippocampal cultures were prepared from embryonic day 18 (E18) embryos of Sprague-Dawley rats as described previously [73] and maintained for 21 days in vitro (DIVs). Purified recombinant Dkk1 (200 ng/mL; PeproTech) was applied to cells for 2 hr in the presence or absence of the Gsk3 inhibitor BIO (200 nM; BioVision Technologies) and ROCK inhibitor Y27632 (10 μM; Selleckchem). See the Supplemental Experimental Procedures for further details.

Immunofluorescence Staining

Brain slices from control and iDkk1 mice were incubated in blocking solution (10% donkey serum and 0.02% v/v Triton X-100 in PBS) for 4 hr at room temperature (RT). Primary antibodies were incubated overnight at 4°C. Secondary antibodies conjugated with Alexa 488, 568, or 647 (1:600; Invitrogen) were incubated at RT for 2 hr. In some experiments, brain sections were incubated with Hoescht for 5 min. Samples were washed in PBS and mounted in Fluoromount-G (SouthernBiotech).

Hippocampal neurons were fixed in 4% paraformaldehyde (PFA) in PBS for 20 min at RT, permeabilized for 5 min in 0.05% v/v Triton X-100 in PBS, and blocked in 5% BSA for 1 hr. Primary antibodies and secondary antibodies were each incubated for 1 hr at RT. Samples were washed in PBS and mounted in FluorSave Reagent (Millipore). See the Supplemental Experimental Procedures for more details.

Image Acquisition and Analyses

For evaluation of synaptic puncta, stacks of eight equidistant planes (0.2 μm; 76 × 76 nm/pixel) from hippocampal slices and cultured neurons were acquired on an Olympus FV1000 confocal microscope using a 60 × 1.35 numerical aperture (NA) oil objective. Four to seven fields were taken per brain slice, and three to four slices were analyzed per mouse. For hippocampal neurons, eight to ten image stacks of EGFP-transfected cells were taken per condition. Analysis was performed in Volocity software (PerkinElmer). See the Supplemental Experimental Procedures for more details.

Electrophysiology

For field potential recordings, parallel bipolar stimulation electrodes were placed in the stratum radiatum of the CA1 region and Schaeffer collateral fibers were stimulated with 0.1 ms duration constant-current paired-pulses (pulse interval 50 ms) delivered to the pathway at intervals of 10 s. Stimulus current was adjusted at the beginning of each recording to give a response approximately 50% of the maximum fEPSPs slope, after recording an input-output curve. fEPSPs were monitored using low-resistance glass pipettes (1–2 MΩ), filled with 4 mM NaCl in ACSF. Slices were subjected to a 15–20 min period of pre-LTP/pre-LTD baseline measurement every 10 s. Provided that the control response did not change by more than 5% during this 15–20 min period, LTP or LTD was induced. LTP was induced by a TBS protocol, which involved delivering two TBSs at an interval of 10 s, and each TBS was composed of five trains of stimuli at intervals of 200 ms, where each train contained four stimuli at 100 Hz. Two protocols of LFS consisting of two trains of 900 pulses delivered at 2 Hz with a 2.5 min gap (strong LFS) or one train of 900 pulses delivered at 2 Hz (weak LFS) were used to induce a LTD. Stimulus intensity for the TBS and LFS was the same as baseline recordings. Paired-pulse fEPSPs (20 Hz) were recorded at intervals of 10 s for at least 50 min after delivery of the TBS or LFS, and the slope of each fEPSP was measured. fEPSP-PPR was calculated as the ratio of the slope of the second to the first fEPSP. Recordings were made using an Axopatch 200B amplifier, filtered (1 kHz) and digitized (10 kHz), and then analyzed using WinEDR software or WinWCP software (freely available at http://spider.science.strath.ac.uk/sipbs/software_ses.htm). For these experiments and patch-clamp recordings, see the Supplemental Experimental Procedures for further information.

Behavioral Studies

For all behavioral tests, adult male mice were handled daily for approximately 2 min, at least 4 days before the beginning of the test. Throughout experimentation and data analysis, the experimenter was blind to genotype. MWM, contextual fear conditioning, T-maze spontaneous alternation, open field, and elevated plus maze tasks are described in the Supplemental Experimental Procedures.

Statistical Analyses

For behavioral analyses, each mouse group consisted of at least seven animals. For immunofluorescence, data were generated from three or more independent experiments, each with one to four mice per genotype. All results were expressed as mean ± SEM. Statistical significance was calculated on the basis of a Student's t test, one-way ANOVA, or ANOVA for repeated measures when samples were normally distributed, followed by Scheffe or Bonferroni posteriori comparisons. Mann-Whitney or Kruskal-Wallis tests were used for non-normally distributed data followed by Dunn-Sidak posteriori comparisons (*$p < 0.05$, ***$p \leq 0.001$, **$p \leq 0.01$).

SUPPLEMENTAL INFORMATION

Supplemental Information includes Supplemental Experimental Procedures and five figures and can be found with this article online at http://dx.doi.org/10.1016/j.cub.2016.07.024.

AUTHOR CONTRIBUTIONS

P.C.S. conceived the project. All authors contributed to the design of experiments, interpretation of the data, and writing of the manuscript. S.P. performed the initial characterization of the Dkk1 induction in the adult hippocampus. D.L., A.M., and M.P. performed behavioral experiments; D.L., S.G., A.M., and F.M. performed cell biology experiments; and A.M. and M.S.-R. performed the electrophysiological recordings. F.C. contributed to design and analysis of behavioral assays. P.C.S. and A.G. provided funding and supervised the project.

ACKNOWLEDGMENTS

We thank Drs. Isabel Mansuy and Sarah E. Millar for transgenic mice and Drs. Elaine E. Irvine for advice on fear conditioning test and E. Stamatakou for breeding and genotyping. We thank Professor John O'Keefe for advice on behavioral tests, Professors Timothy Bliss and Graham Collingridge for useful discussions on LTP and LTD experiments, Drs. Richard Killick and Deepak Srivastava for sharing unpublished results, and Drs. David Attwell and Antonella Riccio and members of the lab for comments on the manuscript. This work was supported by the EU FP7, MRC, ARUK, Wellcome Trust, and Parkinson's UK.

Received: May 24, 2016
Revised: July 5, 2016
Accepted: July 12, 2016
Published: September 1, 2016

REFERENCES

1. Shankar, G.M., and Walsh, D.M. (2009). Alzheimer's disease: synaptic dysfunction and Abeta. Mol. Neurodegener. *4*, 48.
2. Arendt, T. (2009). Synaptic degeneration in Alzheimer's disease. Acta Neuropathol. *118*, 167–179.
3. Budnik, V., and Salinas, P.C. (2011). Wnt signaling during synaptic development and plasticity. Curr. Opin. Neurobiol. *21*, 151–159.
4. Inestrosa, N.C., and Arenas, E. (2010). Emerging roles of Wnts in the adult nervous system. Nat. Rev. Neurosci. *11*, 77–86.
5. Gogolla, N., Galimberti, I., Deguchi, Y., and Caroni, P. (2009). Wnt signaling mediates experience-related regulation of synapse numbers and mossy fiber connectivities in the adult hippocampus. Neuron *62*, 510–525.
6. Kuwabara, T., Hsieh, J., Muotri, A., Yeo, G., Warashina, M., Lie, D.C., Moore, L., Nakashima, K., Asashima, M., and Gage, F.H. (2009). Wnt-mediated activation of NeuroD1 and retro-elements during adult neurogenesis. Nat. Neurosci. *12*, 1097–1105.
7. Ciani, L., Marzo, A., Boyle, K., Stamatakou, E., Lopes, D.M., Anane, D., McLeod, F., Rosso, S.B., Gibb, A., and Salinas, P.C. (2015). Wnt signalling tunes neurotransmitter release by directly targeting Synaptotagmin-1. Nat. Commun. *6*, 8302.
8. De Ferrari, G.V., Papassotiropoulos, A., Biechele, T., Wavrant De-Vrieze, F., Avila, M.E., Major, M.B., Myers, A., Sáez, K., Henríquez, J.P., Zhao, A., et al. (2007). Common genetic variation within the low-density lipoprotein receptor-related protein 6 and late-onset Alzheimer's disease. Proc. Natl. Acad. Sci. USA *104*, 9434–9439.
9. Alarcón, M.A., Medina, M.A., Hu, Q., Avila, M.E., Bustos, B.I., Pérez-Palma, E., Peralta, A., Salazar, P., Ugarte, G.D., Reyes, A.E., et al. (2013). A novel functional low-density lipoprotein receptor-related protein 6 gene alternative splice variant is associated with Alzheimer's disease. Neurobiol. Aging *34*, 1709.e9–1709.e18.
10. Liu, C.C., Tsai, C.W., Deak, F., Rogers, J., Penuliar, M., Sung, Y.M., Maher, J.N., Fu, Y., Li, X., Xu, H., et al. (2014). Deficiency in LRP6-mediated Wnt signaling contributes to synaptic abnormalities and amyloid pathology in Alzheimer's disease. Neuron *84*, 63–77.
11. Mao, B., Wu, W., Li, Y., Hoppe, D., Stannek, P., Glinka, A., and Niehrs, C. (2001). LDL-receptor-related protein 6 is a receptor for Dickkopf proteins. Nature *411*, 321–325.
12. Niehrs, C. (2006). Function and biological roles of the Dickkopf family of Wnt modulators. Oncogene *25*, 7469–7481.
13. Caricasole, A., Copani, A., Caraci, F., Aronica, E., Rozemuller, A.J., Caruso, A., Storto, M., Gaviraghi, G., Terstappen, G.C., and Nicoletti, F.

(2004). Induction of Dickkopf-1, a negative modulator of the Wnt pathway, is associated with neuronal degeneration in Alzheimer's brain. J. Neurosci. *24*, 6021–6027.

14. Rosi, M.C., Luccarini, I., Grossi, C., Fiorentini, A., Spillantini, M.G., Prisco, A., Scali, C., Gianfriddo, M., Caricasole, A., Terstappen, G.C., and Casamenti, F. (2010). Increased Dickkopf-1 expression in transgenic mouse models of neurodegenerative disease. J. Neurochem. *112*, 1539–1551.
15. Bayod, S., Felice, P., Andrés, P., Rosa, P., Camins, A., Pallàs, M., and Canudas, A.M. (2015). Downregulation of canonical Wnt signaling in hippocampus of SAMP8 mice. Neurobiol. Aging *36*, 720–729.
16. Killick, R., Ribe, E.M., Al-Shawi, R., Malik, B., Hooper, C., Fernandes, C., Dobson, R., Nolan, P.M., Lourdusamy, A., Furney, S., et al. (2014). Clusterin regulates β-amyloid toxicity via Dickkopf-1-driven induction of the wnt-PCP-JNK pathway. Mol. Psychiatry *19*, 88–98.
17. Purro, S.A., Dickins, E.M., and Salinas, P.C. (2012). The secreted Wnt antagonist Dickkopf-1 is required for amyloid β-mediated synaptic loss. J. Neurosci. *32*, 3492–3498.
18. Cissé, M., Halabisky, B., Harris, J., Devidze, N., Dubal, D.B., Sun, B., Orr, A., Lotz, G., Kim, D.H., Hamto, P., et al. (2011). Reversing EphB2 depletion rescues cognitive functions in Alzheimer model. Nature *469*, 47–52.
19. De Rosa, R., Garcia, A.A., Braschi, C., Capsoni, S., Maffei, L., Berardi, N., and Cattaneo, A. (2005). Intranasal administration of nerve growth factor (NGF) rescues recognition memory deficits in AD11 anti-NGF transgenic mice. Proc. Natl. Acad. Sci. USA *102*, 3811–3816.
20. Nagahara, A.H., Merrill, D.A., Coppola, G., Tsukada, S., Schroeder, B.E., Shaked, G.M., Wang, L., Blesch, A., Kim, A., Conner, J.M., et al. (2009). Neuroprotective effects of brain-derived neurotrophic factor in rodent and primate models of Alzheimer's disease. Nat. Med. *15*, 331–337.
21. Bie, B., Wu, J., Yang, H., Xu, J.J., Brown, D.L., and Naguib, M. (2014). Epigenetic suppression of neuroligin 1 underlies amyloid-induced memory deficiency. Nat. Neurosci. *17*, 223–231.
22. Galli, S., Lopes, D.M., Ammari, R., Kopra, J., Millar, S.E., Gibb, A., and Salinas, P.C. (2014). Deficient Wnt signalling triggers striatal synaptic degeneration and impaired motor behaviour in adult mice. Nat. Commun. *5*, 4992.
23. Ciani, L., and Salinas, P.C. (2005). WNTs in the vertebrate nervous system: from patterning to neuronal connectivity. Nat. Rev. Neurosci. *6*, 351–362.
24. Park, M., and Shen, K. (2012). WNTs in synapse formation and neuronal circuitry. EMBO J. *31*, 2697–2704.
25. Salinas, P.C. (2012). Wnt signaling in the vertebrate central nervous system: from axon guidance to synaptic function. Cold Spring Harb. Perspect. Biol. *4*, a008003.
26. Chu, E.Y., Hens, J., Andl, T., Kairo, A., Yamaguchi, T.P., Brisken, C., Glick, A., Wysolmerski, J.J., and Millar, S.E. (2004). Canonical WNT signaling promotes mammary placode development and is essential for initiation of mammary gland morphogenesis. Development *131*, 4819–4829.
27. Michalon, A., Koshibu, K., Baumgärtel, K., Spirig, D.H., and Mansuy, I.M. (2005). Inducible and neuron-specific gene expression in the adult mouse brain with the rtTA2S-M2 system. Genesis *43*, 205–212.
28. Mansuy, I.M., Winder, D.G., Moallem, T.M., Osman, M., Mayford, M., Hawkins, R.D., and Kandel, E.R. (1998). Inducible and reversible gene expression with the rtTA system for the study of memory. Neuron *21*, 257–265.
29. Busceti, C.L., Biagioni, F., Aronica, E., Riozzi, B., Storto, M., Battaglia, G., Giorgi, F.S., Gradini, R., Fornai, F., Caricasole, A., et al. (2007). Induction of the Wnt inhibitor, Dickkopf-1, is associated with neurodegeneration related to temporal lobe epilepsy. Epilepsia *48*, 694–705.
30. Seib, D.R., Corsini, N.S., Ellwanger, K., Plaas, C., Mateos, A., Pitzer, C., Niehrs, C., Celikel, T., and Martin-Villalba, A. (2013). Loss of Dickkopf-1 restores neurogenesis in old age and counteracts cognitive decline. Cell Stem Cell *12*, 204–214.
31. von Bohlen Und Halbach, O. (2007). Immunohistological markers for staging neurogenesis in adult hippocampus. Cell Tissue Res. *329*, 409–420.
32. Bannerman, D.M., Rawlins, J.N., McHugh, S.B., Deacon, R.M., Yee, B.K., Bast, T., Zhang, W.N., Pothuizen, H.H., and Feldon, J. (2004). Regional dissociations within the hippocampus–memory and anxiety. Neurosci. Biobehav. Rev. *28*, 273–283.
33. Bannerman, D.M., Sprengel, R., Sanderson, D.J., McHugh, S.B., Rawlins, J.N., Monyer, H., and Seeburg, P.H. (2014). Hippocampal synaptic plasticity, spatial memory and anxiety. Nat. Rev. Neurosci. *15*, 181–192.
34. Lalonde, R. (2002). The neurobiological basis of spontaneous alternation. Neurosci. Biobehav. Rev. *26*, 91–104.
35. Morris, R.G., Garrud, P., Rawlins, J.N., and O'Keefe, J. (1982). Place navigation impaired in rats with hippocampal lesions. Nature *297*, 681–683.
36. Vorhees, C.V., and Williams, M.T. (2006). Morris water maze: procedures for assessing spatial and related forms of learning and memory. Nat. Protoc. *1*, 848–858.
37. Sharma, S., Rakoczy, S., and Brown-Borg, H. (2010). Assessment of spatial memory in mice. Life Sci. *87*, 521–536.
38. Fowler, S.W., Chiang, A.C., Savjani, R.R., Larson, M.E., Sherman, M.A., Schuler, D.R., Cirrito, J.R., Lesné, S.E., and Jankowsky, J.L. (2014). Genetic modulation of soluble Aβ rescues cognitive and synaptic impairment in a mouse model of Alzheimer's disease. J. Neurosci. *34*, 7871–7885.
39. Hochgräfe, K., Sydow, A., and Mandelkow, E.M. (2013). Regulatable transgenic mouse models of Alzheimer disease: onset, reversibility and spreading of Tau pathology. FEBS J. *280*, 4371–4381.
40. Xia, D., Watanabe, H., Wu, B., Lee, S.H., Li, Y., Tsvetkov, E., Bolshakov, V.Y., Shen, J., and Kelleher, R.J., 3rd. (2015). Presenilin-1 knockin mice reveal loss-of-function mechanism for familial Alzheimer's disease. Neuron *85*, 967–981.
41. Phillips, R.G., and LeDoux, J.E. (1994). Lesions of the dorsal hippocampal formation interfere with background but not foreground contextual fear conditioning. Learn. Mem. *1*, 34–44.
42. Maren, S., Phan, K.L., and Liberzon, I. (2013). The contextual brain: implications for fear conditioning, extinction and psychopathology. Nat. Rev. Neurosci. *14*, 417–428.
43. Martin, S.J., Grimwood, P.D., and Morris, R.G. (2000). Synaptic plasticity and memory: an evaluation of the hypothesis. Annu. Rev. Neurosci. *23*, 649–711.
44. Nabavi, S., Fox, R., Proulx, C.D., Lin, J.Y., Tsien, R.Y., and Malinow, R. (2014). Engineering a memory with LTD and LTP. Nature *511*, 348–352.
45. Takeuchi, T., Duszkiewicz, A.J., and Morris, R.G. (2013). The synaptic plasticity and memory hypothesis: encoding, storage and persistence. Philos. Trans. R. Soc. Lond. B Biol. Sci. *369*, 20130288.
46. Larson, J., Wong, D., and Lynch, G. (1986). Patterned stimulation at the theta frequency is optimal for the induction of hippocampal long-term potentiation. Brain Res. *368*, 347–350.
47. McNaughton, B.L., Douglas, R.M., and Goddard, G.V. (1978). Synaptic enhancement in fascia dentata: cooperativity among coactive afferents. Brain Res. *157*, 277–293.
48. Chen, X., Lin, R., Chang, L., Xu, S., Wei, X., Zhang, J., Wang, C., Anwyl, R., and Wang, Q. (2013). Enhancement of long-term depression by soluble amyloid β protein in rat hippocampus is mediated by metabotropic glutamate receptor and involves activation of p38MAPK, STEP and caspase-3. Neuroscience *253*, 435–443.
49. Li, S., Hong, S., Shepardson, N.E., Walsh, D.M., Shankar, G.M., and Selkoe, D. (2009). Soluble oligomers of amyloid Beta protein facilitate hippocampal long-term depression by disrupting neuronal glutamate uptake. Neuron *62*, 788–801.
50. Otani, S., and Connor, J.A. (1996). A novel synaptic interaction underlying induction of long-term depression in the area CA1 of adult rat hippocampus. J. Physiol. *492*, 225–230.
51. Clevers, H., and Nusse, R. (2012). Wnt/β-catenin signaling and disease. Cell *149*, 1192–1205.
52. Meijer, L., Skaltsounis, A.L., Magiatis, P., Polychronopoulos, P., Knockaert, M., Leost, M., Ryan, X.P., Vonica, C.A., Brivanlou, A., Dajani,

R., et al. (2003). GSK-3-selective inhibitors derived from Tyrian purple indirubins. Chem. Biol. *10*, 1255–1266.

53. Purro, S.A., Ciani, L., Hoyos-Flight, M., Stamatakou, E., Siomou, E., and Salinas, P.C. (2008). Wnt regulates axon behavior through changes in microtubule growth directionality: a new role for adenomatous polyposis coli. J. Neurosci. *28*, 8644–8654.
54. Krause, U., Ryan, D.M., Clough, B.H., and Gregory, C.A. (2014). An unexpected role for a Wnt-inhibitor: Dickkopf-1 triggers a novel cancer survival mechanism through modulation of aldehyde-dehydrogenase-1 activity. Cell Death Dis. *5*, e1093.
55. Endo, Y., Beauchamp, E., Woods, D., Taylor, W.G., Toretsky, J.A., Uren, A., and Rubin, J.S. (2008). Wnt-3a and Dickkopf-1 stimulate neurite outgrowth in Ewing tumor cells via a Frizzled3- and c-Jun N-terminal kinase-dependent mechanism. Mol. Cell. Biol. *28*, 2368–2379.
56. Caneparo, L., Huang, Y.L., Staudt, N., Tada, M., Ahrendt, R., Kazanskaya, O., Niehrs, C., and Houart, C. (2007). Dickkopf-1 regulates gastrulation movements by coordinated modulation of Wnt/beta catenin and Wnt/PCP activities, through interaction with the Dally-like homolog Knypek. Genes Dev. *21*, 465–480.
57. Pozueta, J., Lefort, R., Ribe, E.M., Troy, C.M., Arancio, O., and Shelanski, M. (2013). Caspase-2 is required for dendritic spine and behavioural alterations in J20 APP transgenic mice. Nat. Commun. *4*, 1939.
58. Petratos, S., Li, Q.X., George, A.J., Hou, X., Kerr, M.L., Unabia, S.E., Hatzinisiriou, I., Maksel, D., Aguilar, M.I., and Small, D.H. (2008). The beta-amyloid protein of Alzheimer's disease increases neuronal CRMP-2 phosphorylation by a Rho-GTP mechanism. Brain *131*, 90–108.
59. Sfakianos, M.K., Eisman, A., Gourley, S.L., Bradley, W.D., Scheetz, A.J., Settleman, J., Taylor, J.R., Greer, C.A., Williamson, A., and Koleske, A.J. (2007). Inhibition of Rho via Arg and p190RhoGAP in the postnatal mouse hippocampus regulates dendritic spine maturation, synapse and dendrite stability, and behavior. J. Neurosci. *27*, 10982–10992.
60. Cappuccio, I., Calderone, A., Busceti, C.L., Biagioni, F., Pontarelli, F., Bruno, V., Storto, M., Terstappen, G.T., Gaviraghi, G., Fornai, F., et al. (2005). Induction of Dickkopf-1, a negative modulator of the Wnt pathway, is required for the development of ischemic neuronal death. J. Neurosci. *25*, 2647–2657.
61. Mastroiacovo, F., Busceti, C.L., Biagioni, F., Moyanova, S.G., Meisler, M.H., Battaglia, G., Caricasole, A., Bruno, V., and Nicoletti, F. (2009). Induction of the Wnt antagonist, Dickkopf-1, contributes to the development of neuronal death in models of brain focal ischemia. J. Cereb. Blood Flow Metab. *29*, 264–276.
62. Ciani, L., Boyle, K.A., Dickins, E., Sahores, M., Anane, D., Lopes, D.M., Gibb, A.J., and Salinas, P.C. (2011). Wnt7a signaling promotes dendritic spine growth and synaptic strength through Ca^{2+}/Calmodulin-dependent protein kinase II. Proc. Natl. Acad. Sci. USA *108*, 10732–10737.
63. Cuitino, L., Godoy, J.A., Farías, G.G., Couve, A., Bonansco, C., Fuenzalida, M., and Inestrosa, N.C. (2010). Wnt-5a modulates recycling of functional GABAA receptors on hippocampal neurons. J. Neurosci. *30*, 8411–8420.
64. Davis, E.K., Zou, Y., and Ghosh, A. (2008). Wnts acting through canonical and noncanonical signaling pathways exert opposite effects on hippocampal synapse formation. Neural Dev. *3*, 32.
65. Sharma, K., Choi, S.Y., Zhang, Y., Nieland, T.J., Long, S., Li, M., and Huganir, R.L. (2013). High-throughput genetic screen for synaptogenic factors: identification of LRP6 as critical for excitatory synapse development. Cell Rep. *5*, 1330–1341.
66. Liu, J., Zhang, Y., Xu, R., Du, J., Hu, Z., Yang, L., Chen, Y., Zhu, Y., and Gu, L. (2013). PI3K/Akt-dependent phosphorylation of GSK3β and activation of RhoA regulate Wnt5a-induced gastric cancer cell migration. Cell. Signal. *25*, 447–456.
67. Fortress, A.M., Schram, S.L., Tuscher, J.J., and Frick, K.M. (2013). Canonical Wnt signaling is necessary for object recognition memory consolidation. J. Neurosci. *33*, 12619–12626.
68. Bailey, C.H., and Kandel, E.R. (1993). Structural changes accompanying memory storage. Annu. Rev. Physiol. *55*, 397–426.
69. Ge, Y., Dong, Z., Bagot, R.C., Howland, J.G., Phillips, A.G., Wong, T.P., and Wang, Y.T. (2010). Hippocampal long-term depression is required for the consolidation of spatial memory. Proc. Natl. Acad. Sci. USA *107*, 16697–16702.
70. Morris, R.G., Anderson, E., Lynch, G.S., and Baudry, M. (1986). Selective impairment of learning and blockade of long-term potentiation by an N-methyl-D-aspartate receptor antagonist, AP5. Nature *319*, 774–776.
71. Hooper, C., Markevich, V., Plattner, F., Killick, R., Schofield, E., Engel, T., Hernandez, F., Anderton, B., Rosenblum, K., Bliss, T., et al. (2007). Glycogen synthase kinase-3 inhibition is integral to long-term potentiation. Eur. J. Neurosci. *25*, 81–86.
72. Peineau, S., Taghibiglou, C., Bradley, C., Wong, T.P., Liu, L., Lu, J., Lo, E., Wu, D., Saule, E., Bouschet, T., et al. (2007). LTP inhibits LTD in the hippocampus via regulation of GSK3beta. Neuron *53*, 703–717.
73. Dotti, C.G., Sullivan, C.A., and Banker, G.A. (1988). The establishment of polarity by hippocampal neurons in culture. J. Neurosci. *8*, 1454–1468.

Current Biology, Volume *26*

Supplemental Information

Reversal of Synapse Degeneration by Restoring Wnt Signaling in the Adult Hippocampus

Aude Marzo, Soledad Galli, Douglas Lopes, Faye McLeod, Marina Podpolny, Margarita Segovia-Roldan, Lorenza Ciani, Silvia Purro, Francesca Cacucci, Alasdair Gibb, and Patricia C. Salinas

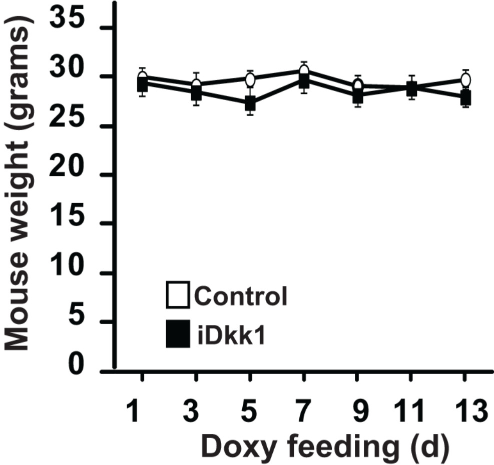
Mouse weight (grams)
35
30
25
20
15
10
5
0
Control
iDkk1
1
3
5
7
9
11
13
Doxy feeding (d)

Figure S1

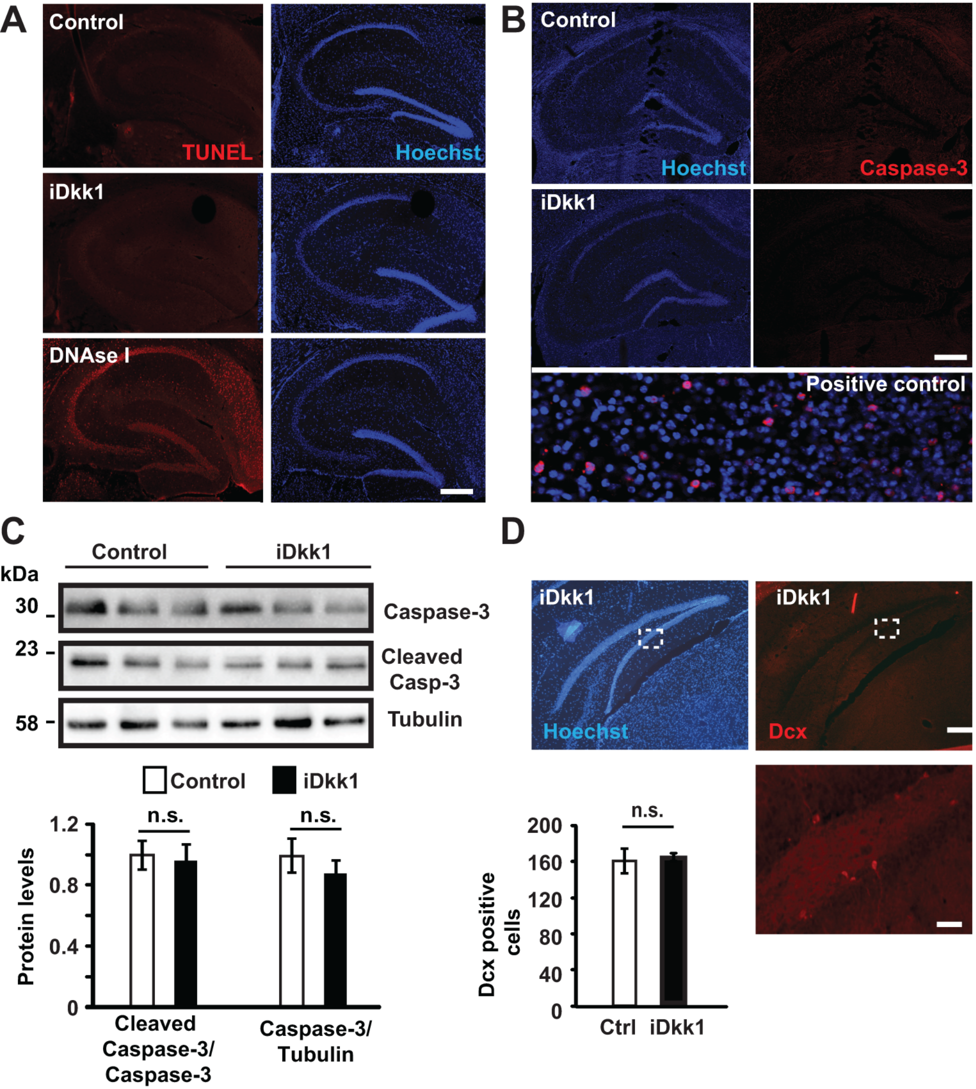
A
Control
TUNEL
Hoechst
iDkk1
DNAse I
B
Control
Hoechst
Caspase-3
iDkk1
Positive control
C
Control
iDkk1
kDa
30
23
58
Caspase-3
Cleaved
Casp-3
Tubulin
Control
iDkk1
1.2
0.8
0.4
0
n.s.
n.s.
Protein levels
Cleaved
Caspase-3/
Caspase-3
Caspase-3/
Tubulin
D
iDkk1
Hoechst
iDkk1
Dcx
200
160
120
80
40
0
n.s.
Dcx positive
cells
Ctrl
iDkk1

Figure S2

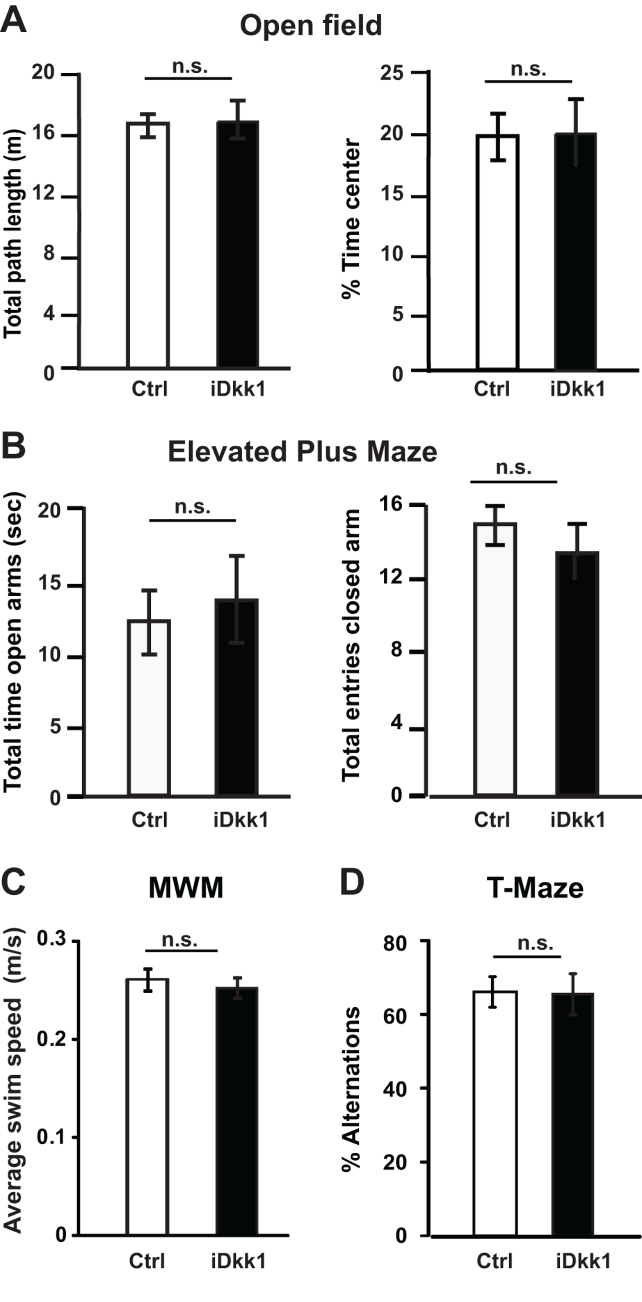
A
Open field
n.s.
Total path length (m)
20
16
12
8
4
0
Ctrl
iDkk1
n.s.
% Time center
25
20
15
10
5
0
Ctrl
iDkk1
B
Elevated Plus Maze
n.s.
Total time open arms (sec)
20
15
10
5
0
Ctrl
iDkk1
n.s.
Total entries closed arm
16
12
8
4
0
Ctrl
iDkk1
C
MWM
n.s.
Average swim speed (m/s)
0.3
0.2
0.1
0
Ctrl
iDkk1
D
T-Maze
n.s.
% Alternations
80
60
40
20
0
Ctrl
iDkk1

Figure S3

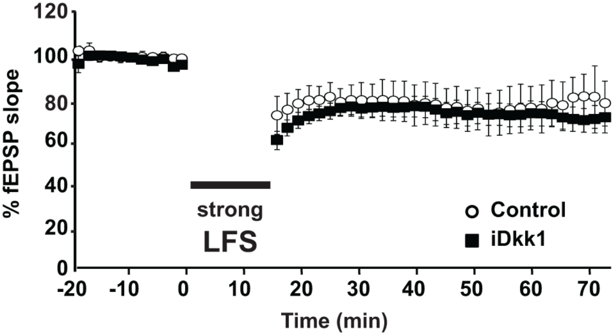

120
100
80
60
40
20
0
% fEPSP slope
strong
LFS
Control
iDkk1
-20
-10
0
10
20
30
40
50
60
70
Time (min)

Figure S4

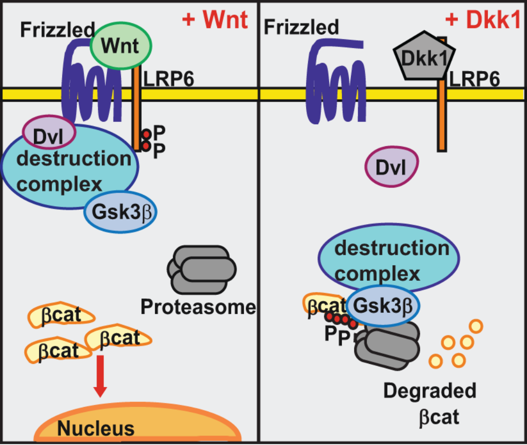
+ Wnt
Frizzled
Wnt
LRP6
P
P
Dvl
destruction complex
Gsk3β
Proteasome
βcat
βcat
βcat
Nucleus
+ Dkk1
Frizzled
Dkk1
LRP6
Dvl
destruction complex
βcat
Gsk3β
P
P
Degraded βcat

Figure S5

Supplemental Legends

Figure S1, refers to Figure 1A: Dkk1 does affect food intake. Weight of control and iDkk1 mice monitored during doxycycline administration. Data are mean ± SEM.

Figure S2, refers to Figure 1D: Dkk1 does not affect overall hippocampus morphology, cell viability and adult neurogenesis in iDkk1 mice. (A) TUNEL assay shows no difference in cell death. DNAse I was used as a positive control. Scale bar: 250 μm. (B) Cleaved caspase 3 staining reveals the level of cell death in the hippocampus. Scale bar: 250 μm. Positive control corresponds to cortical slices from P2 mice. (C) Western blot from hippocampus lysates shows no changes in the levels of caspase-3 activation in iDkk1 mice. Graph demonstrates that levels of cleaved caspase-3 in relation to uncleaved caspase-3 are equal in both groups. Uncleaved caspase-3 levels are unaffected in iDkk1 mice. (Student's *t*-test, 3 animals per genotype). (D) Images and quantification of newborn neurons labelled with Doublecortin (Dcx) in the dentate gyrus from iDkk1 mice (Student's *t*-test, 4 mice per genotype). Scale bar: 100 μm in panoramic view and 50 μm in high magnification images. Data are presented as mean ± SEM.

Figure S3, refers to Figure 1: Induced expression of Dkk1 does not affect anxiety-like behavior in iDkk1 mice. (A) Total path length in the Open Field (left panel) and percentage of time spent in the center of the open field arena (right panel; Student's *t*-test). (B) Time spent in the open arms of the elevated plus maze (left panel) and total number of entries to the closed arms of the elevated plus maze (right panel; Student's *t*-test, 15 control and 11 iDkk1 mice). (C) Average swimming speed in the Morris Water Maze (MWM, Student's *t*-test). (D) Percentage of alternation in the T-maze test (15 control and 10 iDkk1 mice, *$p<0.05$, Student's *t*-test). Results are shown as mean ± SEM.

Figure S4, refers to Figure 2: Dkk1 expression does not alter LTD induced by a strong LFS. LTD induced by delivery of a strong LFS, starting at time zero (8 slices from 7 control (open circles) and 14 slices from 9 iDkk1 mice (black squares); repeated measures ANOVA). Data are presented as mean ± SEM.

Figure S5, refers to Figure 6: Illustration of the Wnt canonical pathway. Binding of Wnt to Frizzled (Fz) and LRP6 receptors recruits Dishevelled (Dvl) resulting in inhibition of Gsk3β-mediated phosphorylation of β-catenin (β-cat) promoting its stabilization (left panel). In contrast, Dkk1 binding to LRP6 (right panel) prevents the formation of the Wnt/Fz/LRP6 complex, thus inhibiting the canonical Wnt pathway resulting in the activation of Gsk3β and in the degradation of β-catenin by the proteasome.

Supplemental Experimental Procedures

Animals

Double transgenic mice (iDkk1) were obtained by crossing tetO-Dkk1 transgenic mice [S1] with CaMKIIα-rtTA2 transgenic mice [S2]. TetO-Dkk1 transgenic mice and CaMKIIα-rtTA2 were crossed in heterozygous state. Both mouse lines were bred in a C57BL/6J background for at least 6 generations. Genotyping was performed using DNA from ear biopsies, using the following primers: CaMKIIα-rtTA: forward 5' TGCCTTTCTCTCCACAGGTGTCC 3' and reverse 5' GAGAGCACAGCGGAATGAC 3'; tetO-Dkk1: forward 5' GCGTCCTTCGGAGATGATGG 3', and reverse 5' AAATGGCTGTGGTCAGAGGG 3'.

Hippocampal culture, cell transfection and drug treatment

High-density hippocampal cultures (250 cells/mm^2) were transfected at 7-8 DIV using calcium phosphate with EGFP-actin to visualize dendrites and spines.

RT-PCR analyses

RNA was extracted from the hippocampus of 3 adult mice (3 months old) using Trizol (Invitrogen) and treated with DNAse I (Sigma). First, strand complementary DNA synthesis was performed with AMV Reverse Transcriptase (Promega) according to the manufacturer's instruction. PCR was performed using GoTaq Polymerase (Promega). The primers used are forward 5' ATTCCAACGCGATCAAGAAC 3' and reverse 5' GCTTGGTGCATACCTGACCT 3'.

In situ hybridization

In situ hybridization was performed as previously reported [S3]. Brains were snap-frozen in pre-cooled isopentane and kept at –80°C. Sagittal sections (12 μm), cut in a cryostat, were air-dried and fixed in 4% paraformaldehyde (PFA) in PBS for 20 min. Slides were incubated with anti-sense or sense Dkk1 cRNA probe [S4] prepared using the DIG-RNA labelling kit (Invitrogen), overnight at 55° C. Slides were then washed and incubated with goat anti-DIG-AP for 3.5 hours. Following washes, samples were incubated with nitro-blue

tetrazolium (NBT) and 5-bromo-4-chloro-3'-indolyphosphate (BCIP) for chromogenic detection, washed in TE buffer to stop the reaction and mounted in Fluoromount-G (SouthernBiotech).

Preparation of brain sections

Brains from control and iDkk1 mice were rapidly dissected and placed in artificial cerebrospinal fluid (ACSF) containing in mM: 126 NaCl, 3 KCl, 2 $CaCl_2$, 1 $MgCl_2$, 1.25 NaH_2PO_4, 26 $NaHCO_3$, 10 D-glucose, pH 7.4. For cleaved caspase 3, NeuN and Doublecortin staining, brains from perfused mice were post-fixed overnight (4% PFA), immerged in 30% sucrose/PBS and frozen in pre-cooled isopentane. Sections (30 µm) were collected onto Superfrost Plus–VWR slides and stored at -20 °C.

Immunofluorescence staining

List of primary antibodies used: vGlut1 (1:2000; Millipore), PSD95 (1:500; Thermo Scientific), Homer1 (1:1000; Synaptic Systems), vGat (1:500; Synaptic Systems), Gephyrin (1:500; Synaptic Systems), β-catenin (1:500; BD transduction labs), cleaved caspase 3 (1:500, Cell Signaling), NeuN (1:500, Millipore), Doublecortin (1:200; Santa Cruz) and GFP (1:1000; Millipore)

TUNEL staining was performed with ApoptoTag® Red *In Situ* Apoptosis detection kit (Chemicon International). For positive controls, brain slices were treated with 1 µg/mL DNAse I at RT**.** For cleaved caspase-3 experiment, positive control consists on horizontal acute brain slices of cortex from P2 mice were incubated at 37 ° C for a period of 4 h and then stained as above.

Image acquisition and analyses

For NeuN experiments, image stacks of 8 equidistant planes were captured on an Olympus FV1000 confocal microscope using a 20× 1.35 NA oil objective. Four to seven fields of the CA1 pyramidal cell layer were acquired per animal. Positive labelled neurons were manually counted in Volocity software (Perkin Elmer) using maximum projection stacks. For Doublecortin experiments, single plane images were captured on an Olympus BX60 microscope with a 10× 1.3 NA objective. Doublecortin positive cells were counted and analysed as previously described [S5].

Analyses were performed in Volocity software. Pre- and postsynaptic puncta number was determined using customized Volocity protocols based on standard thresholding techniques. For hippocampal cells, three dendrites were cropped from maximum projection images of each cell and synaptic puncta were visualized using threshold protocols and normalized to the length of the dendrite. Synapses were defined as the co-localization between vGlut1 and Homer1 puncta.

Electron microscopy

Samples were prepared as described previously [S6]. Brains were fixed with 4% PFA, 0.5% glutaraldehyde in 0.1 M Milloning phosphate buffer, pH 7.4 overnight at 4°C. Coronal sections (200 µm) were cut on a vibratome and post fixed in 1% osmium tetroxide in cacodylate buffer for 1 h, stained in aqueous uranyl acetate for 45 min, dehydrated in graded alcohol and embedded in resin. Ultra-thin sections (70 nm) of silver-gold interference colour were cut and collected on a 200 mesh-grid. Photographs were taken at 40,000X magnification with a JEOL 1010 microscope. Asymmetric synapses were considered when a clear pre- and postsynaptic membrane was visualized, with the presence of a prominent PSD and vesicles in the pre-synaptic terminal [S7, 8].

Western Blot

Homogenates from hippocampus of iDkk1 and control animals (3-4 mice each) were run on 10% SDS-PAGE gels, and Western blots were probed with antibodies against total caspase-3 (1:1000, Synaptic Systems), cleaved caspase 3 (1:1000, Synaptic Systems) and α-tubulin (1: 2000, Sigma). Measurements of band intensity were performed using ImageJ software (NIH).

Electrophysiology

Brains were quickly dissected and transversal hippocampal slices were collected in high-sucrose-ACSF containing in mM: NaCl 75, $NaHCO_3$ 25, KCl 2.5, $NaHPO_4$ 1.25, kynurenic acid 1.25, pyruvic acid 2, EDTA 0.1, $CaCl_2$ 1, $MgCl_2$ 4, D-glucose 25 and sucrose 100, bubbled with 95% O_2/5% CO_2. Slices were kept at RT for patch clamp recordings or placed at 34°C for 1-2h before recordings for field potential recordings.

For field potential recordings, brain slices (400 µm thick) were placed in a chamber continuously perfused at 30°C with recording solution containing in mM: NaCl 125, $NaHCO_3$ 25, KCl 2.5, NaH_2PO_4 1.25, $CaCl_2$ 2, $MgCl_2$ 1 and D-glucose 25 and bubbled with 95% O_2/5% CO_2.

For patch clamp recordings, brain slices (300 µm thick) were placed in a chamber on an upright microscope and continuously superfused at room temperature with ACSF recording solution (in mM: NaCl 125, $NaHCO_3$ 25, KCl 2.5, $NaHPO_4$ 1.25, $CaCl_2$ 1, and D-glucose 25), bubbled with 95% O_2/5% CO_2. CA1 hippocampal cells were patched in whole cell voltage-clamp configuration using pipettes (resistance 5–8 MΩ) pulled from

borosilicate glass (Harvard GC150F-7.5) and filled with pipette solution containing in mM: D-gluconic acid lactone 139, HEPES 10, EGTA 10, NaCl 10, $CaCl_2$ 0.5, $MgCl_2$ 1, ATP 1 and GTP 1, adjusted to pH 7.2 with CsOH. When recording miniature currents, 100 nM TTX was included in the recording solution. Miniature or evoked EPSCs were recorded at -60 mV in the presence of 10 µM bicuculline and 50 µM AP-5, whereas IPSCs were recorded at 0 mV in the presence of 50 µM AP-5.

Behavioral studies

Morris Water Maze

Control and iDkk1 mice were fed with doxycycline for 5 days before the beginning of the task, and doxycycline feeding was maintained throughout the test. The test was performed in a circular 120 cm diameter pool as previously described [S9] with a few modifications. Briefly, the paradigm consisted of two training phases: 4 days with a visible platform, followed by 5 days (acquisition phase) with a hidden platform. For each training phase, a four trials/day training protocol with 10 min inter-trial intervals and maximum trial duration of 90 sec was used. In the first phase, mice were trained to locate a pseudo-randomly placed platform with a local cue (flag) protruding out of the water surface. At this stage, curtains were drawn around the pool to occlude any distal cues. During the second training phase, mice were trained to find a hidden platform with the extra-maze cues visible around the room. During probe trials platform was removed from the pool and mice were allowed to locate the platform for up to 90 sec. Probe trials were conducted before the fourth day of training (Probe I) and 24 h after the last day of training (Probe II). All trials were recorded with a camera located in the ceiling and images were analyzed using the DacqTrack-WaterMaze tracking system (Axona Ltd.).

Contextual Fear Conditioning

Mice were placed into a conditioning chamber (Med Associates Inc.) located in a soundproof box, equipped with a video camera. The conditioning chamber floor was a stainless steel grid used for shock delivery. A speaker mounted on the wall was used to deliver the tone. The contextual fear conditioning protocol used was as previously described [S10]. Briefly, on the conditioning day, mice were allowed to acclimatize to the room. Mice were then placed individually in the conditioning chamber and after a 120-sec introductory period, a tone (80 dB, 3.0 kHz) was presented for 30 sec, the last 2 sec of which coincided with a foot-shock (0.7 mA). Contextual fear memory was tested 24 h after training by re-exposure to the conditioning chamber for 5 min, with no shock delivery. Freezing behavior (defined as complete lack of movement, except for respiration) was scored for 2 sec every 5 sec by an experimenter blind to the genotype of the mice. Baseline freezing was considered as the levels of freezing prior to the presentation of the stimulus.

T-maze spontaneous alternation

The enclosed T-maze consisted of 2 arms of 27 x 7 x 10 cm each, made of white Formica. A partition extended 7 cm from the back of the T into the start arm. A set of three guillotine doors was used to separate the entrance of each arm. At the beginning of the test all the guillotine doors were raised, except for the one located at the end on the starting arm. Each mouse received eight consecutive trials in the enclosed T-maze. Each trial consisted in putting individually the animal at the entrance of the main arm and allowing the mouse to freely run to the goal arm. After the mouse has entered the goal arm, the guillotine door was closed and the animal was confined in the chosen goal arm for 30 sec. The mouse was then returned to the start partition, where it was held for 30 sec. Results are expressed as the number of correct alternations over the total number of possible correct alternations. Correct alternation was scored whenever the mouse entered into a previously unvisited arm.

Open Field

The apparatus consisted of a 45 x 45 x 45 cm wooden box open at the top, illuminated with a dim light. Mice were placed individually in the center of the arena, in a position equidistant to the walls, and allowed to explore the open field for 300 seconds. Mice were tracked using a video camera fixed to the ceiling of the room and connected to a digital video tracking system (HVS Image Ltd.). Distance travelled and time spent in the central and peripheral areas were monitored by the DacqTrack tracking system (Axona Ltd.).

Plus-maze

The apparatus, made of wood laminated with white formica, consisted of four 30 x 5 cm arms. Two of the arms were surrounded by 15 cm height walls. The apparatus was elevated 40 cm above the floor and lit by dim light. Mice were placed individually in the central area (neutral area) and monitored for 300 sec as described above.

Supplemental References

S1. Chu, E.Y., Hens, J., Andl, T., Kairo, A., Yamaguchi, T.P., Brisken, C., Glick, A., Wysolmerski, J.J., and Millar, S.E. (2004). Canonical WNT signaling promotes mammary placode development and is essential for initiation of mammary gland morphogenesis. Development *131*, 4819-4829.

S2. Michalon, A., Koshibu, K., Baumgartel, K., Spirig, D.H., and Mansuy, I.M. (2005). Inducible and neuron-specific gene expression in the adult mouse brain with the rtTA2S-M2 system. Genesis *43*, 205-212.

S3. Pasterkamp, R.J., De Winter, F., Holtmaat, A.J., and Verhaagen, J. (1998). Evidence for a role of the chemorepellent semaphorin III and its receptor neuropilin-1 in the regeneration of primary olfactory axons. J. Neurosci *18*, 9962-9976.

S4. Andl, T., Reddy, S.T., Gaddapara, T., and Millar, S.E. (2002). WNT signals are required for the initiation of hair follicle development. Dev Cell *2*, 643-653.

S5. Licht, T., Goshen, I., Avital, A., Kreisel, T., Zubedat, S., Eavri, R., Segal, M., Yirmiya, R., and Keshet, E. (2011). Reversible modulations of neuronal plasticity by VEGF. Proc. Natl. Acad. Sci. USA *108*, 5081-5086.

S6. Ciani, L., Marzo, A., Boyle, K., Stamatakou, E., Lopes, D.M., Anane, D., McLeod, F., Rosso, S.B., Gibb, A., and Salinas, P.C. (2015). Wnt signalling tunes neurotransmitter release by directly targeting Synaptotagmin-1. Nat Commun *6*, 8302.

S7. Bourne, J.N., and Harris, K.M. (2008). Balancing structure and function at hippocampal dendritic spines. Annu Rev Neurosci *31*, 47-67.

S8. Schikorski, T., and Stevens, C.F. (1997). Quantitative ultrastructural analysis of hippocampal excitatory synapses. J. Neurosci *17*, 5858-5867.

S9. Malleret, G., Haditsch, U., Genoux, D., Jones, M.W., Bliss, T.V., Vanhoose, A.M., Weitlauf, C., Kandel, E.R., Winder, D.G., and Mansuy, I.M. (2001). Inducible and reversible enhancement of learning, memory, and long-term potentiation by genetic inhibition of calcineurin. Cell *104*, 675-686.

S10. Irvine, E.E., Drinkwater, L., Radwanska, K., Al-Qassab, H., Smith, M.A., O'Brien, M., Kielar, C., Choudhury, A.I., Krauss, S., Cooper, J.D., et al. (2011). Insulin receptor substrate 2 is a negative regulator of memory formation. Learn Mem *18*, 375-383.
